# Comparative Analysis of Three Brevetoxin-Associated Bottlenose Dolphin (*Tursiops truncatus*) Mortality Events in the Florida Panhandle Region (USA)

**DOI:** 10.1371/journal.pone.0042974

**Published:** 2012-08-15

**Authors:** Michael J. Twiner, Leanne J. Flewelling, Spencer E. Fire, Sabrina R. Bowen-Stevens, Joseph K. Gaydos, Christine K. Johnson, Jan H. Landsberg, Tod A. Leighfield, Blair Mase-Guthrie, Lori Schwacke, Frances M. Van Dolah, Zhihong Wang, Teresa K. Rowles

**Affiliations:** 1 Marine Biotoxins Program, National Oceanic and Atmospheric Administration/National Ocean Service, Charleston, South Carolina, United States of America; 2 Department of Natural Sciences, University of Michigan-Dearborn, Dearborn, Michigan, United States of America; 3 Florida Fish and Wildlife Conservation Commission, Fish and Wildlife Research Institute, St. Petersburg, Florida, United States of America; 4 National Oceanic and Atmospheric Administration Fisheries, Southeast Fisheries Science Center, Panama City Laboratory, Panama City, Florida, United States of America; 5 National Oceanic and Atmospheric Administration/National Marine Fisheries Service, Southeast Fisheries Science Center, Miami, Florida, United States of America; 6 Wildlife Health Center, School of Veterinary Medicine, University of California Davis, Davis, California, United States of America; 7 National Oceanic and Atmospheric Administration/National Ocean Service, Cooperative Center for Marine Animal Health, Hollings Marine Laboratory, Charleston, South Carolina, United States of America; 8 National Oceanic and Atmospheric Administration Fisheries, Cooperative Center for Marine Animal Health, Silver Spring, Maryland, United States of America; Texas A&M University-Corpus Christi, United States of America

## Abstract

In the Florida Panhandle region, bottlenose dolphins (Tursiops truncatus) have been highly susceptible to large-scale unusual mortality events (UMEs) that may have been the result of exposure to blooms of the dinoflagellate Karenia brevis and its neurotoxin, brevetoxin (PbTx). Between 1999 and 2006, three bottlenose dolphin UMEs occurred in the Florida Panhandle region. The primary objective of this study was to determine if these mortality events were due to brevetoxicosis. Analysis of over 850 samples from 105 bottlenose dolphins and associated prey items were analyzed for algal toxins and have provided details on tissue distribution, pathways of trophic transfer, and spatial-temporal trends for each mortality event. In 1999/2000, 152 dolphins died following extensive K. brevis blooms and brevetoxin was detected in 52% of animals tested at concentrations up to 500 ng/g. In 2004, 105 bottlenose dolphins died in the absence of an identifiable K. brevis bloom; however, 100% of the tested animals were positive for brevetoxin at concentrations up to 29,126 ng/mL. Dolphin stomach contents frequently consisted of brevetoxin-contaminated menhaden. In addition, another potentially toxigenic algal species, Pseudo-nitzschia, was present and low levels of the neurotoxin domoic acid (DA) were detected in nearly all tested animals (89%). In 2005/2006, 90 bottlenose dolphins died that were initially coincident with high densities of K. brevis. Most (93%) of the tested animals were positive for brevetoxin at concentrations up to 2,724 ng/mL. No DA was detected in these animals despite the presence of an intense DA-producing Pseudo-nitzschia bloom. In contrast to the absence or very low levels of brevetoxins measured in live dolphins, and those stranding in the absence of a K. brevis bloom, these data, taken together with the absence of any other obvious pathology, provide strong evidence that brevetoxin was the causative agent involved in these bottlenose dolphin mortality events.

## Introduction

Harmful algal blooms (HABs) are commonly known for their detrimental impacts on aquatic organisms (including marine mammals), human health, and local economies [1]–[3]. *Karenia brevis* blooms, often referred to as “Florida red tide”, occur in the Gulf of Mexico (Florida, USA) on a nearly annual basis [4]. This dinoflagellate produces a suite of neurotoxins known as brevetoxins (PbTxs or BTXs), which are heat-stable, lipid-soluble, polyether compounds [5]–[7]. The proximate mammalian pharmacological target of brevetoxin(s) is site 5 on the voltage-gated sodium channel [5], where it binds with high affinity (Kd 1–50 nM; [8]). Once bound, these toxins alter the voltage sensitivity of the channel by interfering with the voltage sensor and inactivation gate, ultimately resulting in nerve inhibition [9], [10].

Human health effects from brevetoxins generally result from neurotoxic shellfish poisoning (NSP) [11] and/or respiratory illness caused by inhalation of aerosolized toxin [12]. The former can occur following consumption of contaminated shellfish that have accumulated sufficient levels of toxin while filter feeding the algal assemblage including *K. brevis*. NSP typically affects the nervous and gastrointestinal systems; however, all symptoms are reversible and to date there have been no human associated deaths [3], [11]. Humans can also be exposed to aerosolized brevetoxins after the fragile, unarmored *K. brevis* cells lyse due to wave action, releasing toxins into the air [13] and causing irritation and burning of the throat and upper respiratory tract [14] following inhalation. In addition to brevetoxins acting as potent ichthyotoxins [15], Bossart *et al.* [16] observed brevetoxin immunoreactivity in the lung, liver, and lymphoid tissues of manatees collected during a 1996 mortality event, suggesting that brevetoxins can be absorbed by mammals via the inhalation of aerosolized toxins [16], [17].

Although a comprehensive understanding of brevetoxin trophic transfer is lacking, it is clear that finfish [18]–[20] and certain types of seagrasses (i.e., *Thalassia testudinum*) can accumulate or vector brevetoxins and play a primary role in brevetoxin-induced marine mammal mortality events or strandings [20], [21]. If the mortality event is deemed “a stranding that is unexpected; involves a significant die-off of any marine mammal population; and demands immediate response”, it may be declared an unusual mortality event (UME) under the authority of the Marine Mammal Protection Act (www.nmfs.noaa.gov/pr/laws/mmpa/). Historically, bottlenose dolphin (*Tursiops truncatus*) mass mortality events in the Gulf of Mexico have long been thought to be associated with *K. brevis* [22], with the first documented incident of dolphin mortalities occurring simultaneously with an 8-month long bloom in southwest Florida that began in November 1946 [23]. However, at that time linking the deaths to the *K. brevis* bloom was not possible due to the fact that the relationship between *K. brevis* and brevetoxin had not been identified [11]. Despite all of the advances since this time, the mechanisms that lead to marine mammal death as a result of brevetoxicosis remain unresolved. Given that experimentation on protected species is prohibited, a level that constitutes a lethal dose of brevetoxin for a marine mammal may never be definitively known.

Another HAB toxin found in the Gulf of Mexico is domoic acid (DA), which is produced by members of the diatom genus *Pseudo-nitzschia*. DA is a neurotoxin that can cause amnesic shellfish poisoning (ASP) in humans [24] and large-scale mortality of sea birds [25], pinnipeds [26], [27], and cetaceans [22]. DA is an analog of the neurotransmitter glutamate and a partial agonist that binds with high affinity to kainate receptors and intermediate affinity to α-amino-3-hydroxyl-5-methyl-4-isoxazole-propionate glutamate receptor subunits [28]. Trophic transfer of DA via zooplankton into higher organisms has been well documented in krill [29], shellfish [30], sand crabs [31], and fish [27]. In the Texas region of the Gulf of Mexico, DA was first reported in phytoplankton in 1989 [32] with *Nitzschia pungens* f. *multiseries* (syn. *P. multiseries*) identified as the putative producer of DA [32], [33]. A consortium of *Pseudo-nitzschia* species was subsequently identified (*P. pseudodelicatissima, P. delicatissima, P. multiseries, P. pungens, P. subfraudulenta*) in nearby Louisiana coastal waters [34]. The most dominant species, *P. pseudodelicatissima*, was shown to produce DA *in situ* [34] and in culture [35] at levels that are not unlike *Pseudo-nitzschia* isolates found in other coastal regions susceptible to DA-related UMEs and/or ASP events. A region documented to have relatively high numbers of *Pseudo-nitzschia* spp. has been identified in the northern Gulf of Mexico, near coastal Louisiana, near Mobile Bay, Alabama, and near Perdido Bay, Florida, that appears to be highly influenced by high nutrient, submarine waters [36].

As top predators, bottlenose dolphins are considered sentinels for ecosystem health [37], [38] and studies examining the health of coastal dolphin populations have suggested pathways for transport of brevetoxin through the marine food web. One long-term study of a population in Sarasota Bay, Florida [39] has shown that a major route of exposure to brevetoxin for bottlenose dolphins appears to be consumption of finfish such as pinfish (*Lagodon rhomboides*), pigfish (*Orthopristis chrysoptera*), striped mullet (*Mugil cephalus*), and spot (*Leiostomus xanthurus*) [19], [40], which are among the primary prey items for bottlenose dolphins in the Sarasota Bay region [41], [42]. These dolphins and their prey items are both known to heavily utilize seagrass habitats within the bay, and the dolphins' home ranges and dietary analysis indicate that they are year-round, permanent residents of the inshore bay system and up to 1 km offshore [41], [43]. Although *K. brevis* blooms have been shown to affect fish population diversity and community structure [44], [45], detectable levels of brevetoxin have been identified in shellfish and finfish for up to one year following a *K. brevis* bloom [18], [19], [46], although the absence of cells at bloom concentrations does not necessarily infer the absence of brevetoxin in the system [21]. In contrast to studies involving brevetoxin, there have been few studies examining the presence or effects of DA or DA-producing *Pseudo-nitzschia* species on the bottlenose dolphin population in Sarasota Bay. Nonetheless, a recent study in this population illustrated the chronic exposure and accumulation of DA (concurrent with brevetoxin) in bottlenose dolphins [40]. These exposures appear to correlate with indices of immunomodulation.

Although red tides are almost annual along the central west coast of Florida, bloom events in the Florida Panhandle region of the Gulf of Mexico are less common [4]. From the late 1990s, two red tide events in this region coincided with two bottlenose dolphin UMEs, while in 2004 another bottlenose dolphin UME was not obviously associated with a concurrent red tide bloom. The primary objective of this study therefore was to assess and compare these three distinct bottlenose dolphin UMEs that were putatively associated with brevetoxin and the Florida red tide in 1999/2000, 2004, and 2005/2006. In total, over 850 samples from 105 bottlenose dolphins were analyzed for algal toxins using various detection strategies. For the first time, these data have been used to determine the routes of trophic transfer, extent of exposure and accumulation, and toxin distribution in a protected marine mammal species and its prey from exposure to two lethal algal toxins.

## Materials and Methods

### 2.1 Water collections and cell count data

Before the unusual dolphin mortality of 2004, HAB sampling in the Florida Panhandle was not routine and was mainly initiated in response to discolored water, fish kills, or aquatic animal strandings or mortalities. Since 2004 routine HAB monitoring and HAB research efforts in this region have increased. *K. brevis* and *Pseudo-nitzschia* spp. cell counts reported here were obtained from FWC-FWRI's Harmful Algal Bloom database, which contains statewide HAB data collected for monitoring, research, and event response. The samples were collected by staff from multiple state agencies as well as volunteer members of the FWRI Red Tide Offshore Monitoring Program and local non-profit organizations. FWC-FWRI HAB staff utilized standard protocols for sampling, identifying, and counting HAB species [47], [48]. The detection limit (dl) for each species was 333 cells/L. In a subset of samples, particulate and dissolved brevetoxins in water were analyzed according the methods described by Pierce et al. [49] and Twiner et al. [50].

### 2.2 Stranding data

Level A stranding data associated with dead bottlenose dolphins were collected on location and during post-mortem examinations. Data included geographical location, species identification, gender, estimated length-determined age class, and carcass condition code (ranging from 1 to 5 with 1 live and 5 severely decomposed). In the absence of tooth growth layer data for aging, animal length has been used as proxy for age class and is based on the following scale: neonates included total length up to 137 cm, yearlings were 138–186 cm, juveniles were 187–227 cm, subadults were 228–247 cm, and adults were 248 cm or longer [51], [52]. Due to concerns over carcass decomposition, only code 1–3 animals (code 1 =  live, code 2 =  fresh dead, code 3 =  mild decomposition) were subject to algal toxin analysis.

### 2.3 Bottlenose dolphin carcass and tissue collections

**Figure 1:**
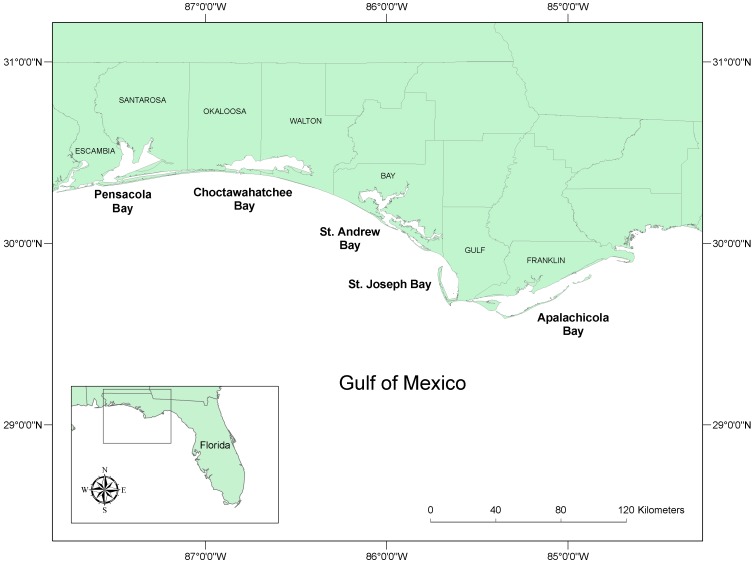
Florida Panhandle region (Gulf of Mexico, USA).

Samples for histopathology, toxicology and other ancillary tests such as microbiology and virology were collected from stranded bottlenose dolphins by members of the Southeast Marine Mammal Stranding Network during all three UMEs along the Florida Panhandle (Figure 1). Algal toxin sampling strategies evolved over this time frame. In 1999–2000, analyses focused on liver, kidney and spleen. However, subsequent to that investigation we found that the samples most useful for the rapid confirmation of algal toxin exposure in marine mammals are stomach contents, urine, and feces (unpublished data); therefore, in 2004 and 2005–2006 these samples were initially screened for the presence of brevetoxin and/or DA. Following the confirmation of either algal toxin, further analyses were carried out to gain insight into their tissue distribution in liver, kidney, spleen, blood, brain, and lung. In 2004 and 2005–2006 blood samples were also collected on blood spot cards [53] in cases where samples were sufficiently fresh that blood was not coagulated. Not all samples were collected from all animals, depending on body condition and the availability of field assistance. All samples were stored frozen at 20°C and shipped to toxin analysis laboratories at the NOAA Marine Biotoxins Program (Charleston, SC) and the Florida Fish and Wildlife Conservation Commission's Fish and Wildlife Research Institute (St. Petersburg, FL; 2004 and 2005–2006 only).

### 2.4 Prey item collections

Various fish species and crustaceans were collected from bottlenose dolphin stomach contents and by cast net in the study areas. Whole fish were identified, sorted according to species, weighed, and frozen at −20°C [54]. Viscera (all organs in intracoelomic cavity), stomach contents, liver, and muscle, were dissected, weighed, and extracted for algal toxins. If the prey item was too small, it was analyzed whole.

### 2.5 Algal toxin analysis

Brevetoxins were the primary algal toxin class investigated based on the presence of *K. brevis* blooms concurrent with the mortality events (i.e., 1999/2000, 2005/2006) [17] or because of their known impacts of marine mammals in the region (i.e., 2004) [20], [55]. For brevetoxin screening assays, we used a competitive enzyme-linked immunosorbent assay (ELISA), a receptor binding assay (RBA), or a radioimmunoassay (RIA). Many of the samples testing positive in one of the screening assays were confirmed by HPLC-tandem mass spectrometry (LC-MS/MS). In the 2004 and 2005/2006 UMEs, DA was also analyzed in the blood, urine, stomach contents, liver, kidney, lung, and muscle samples of stranded animals. An ELISA for DA was used as a rapid screen, followed by LC-MS/MS confirmation of DA on positive samples. Since many samples were analyzed using two or more independent methods, all data are presented as a mean of the independent analyses. The only exception are the data presented in Tables S1, S2, and S3.

#### 2.5.1 Brevetoxin extraction

Urine, gastric fluid, and liquid fecal samples were centrifuged at 13000*×g* for 10 minutes at 25°C, the supernatant was removed and filtered through a 0.45 µm syringe filter prior to analysis to remove particulates, and analyzed without solvent extraction. Solid samples (stomach contents, feces, liver, kidney, spleen, brain, lung) were homogenized and extracted in acetone four times (3 volumes or 3 mL minimum, depending on sample mass: typically 10 grams), filtered through a 0.45 µm syringe filter, dried under nitrogen gas, resuspended in 80% aqueous methanol (6 volumes or 30 mL), twice solvent partitioned with hexane (3 volumes or 30 mL), and the methanolic layer collected, dried under nitrogen gas and resuspended in 100% methanol (0.5 or 5 mL). Extracts were stored at −20°C until analysis. For some of the 2005/2006 samples, we used a modification of this method: 2 grams of sample were extracted twice in 9 mL 80% methanol, each time heated to 60°C for 20 min, combined and brought to 20 mL. An 8 mL aliquot of the extract was then washed with 10 mL hexane and the methanolic layer retained for analysis.

Blood card samples (100 µL blood) were extracted for brevetoxins using 0.8 mL of a phosphate buffered saline amended with (6%) methanol followed by adding 2.4 mL of acetonitrile to precipitate proteins. Samples were centrifuged at 4000*×g* for 15 min at 4°C and the supernatant was removed for analysis.

#### 2.5.2 Brevetoxin analysis by receptor binding assay (RBA)

Clarified or extracted urine, feces, liver, gastric fluid, and stomach content samples were analyzed using a brevetoxin RBA. The RBA is a functional bioassay in which an unknown quantity of non-radiolabled brevetoxin (standards or samples) competes with radiolabeled brevetoxin (^3^H-PbTx-3; Amersham, NJ, USA) for the site 5 receptor of the voltage-gated sodium channel [50], [56]. Data are expressed as PbTx-3 equivalents (equiv.), representing the toxic potency of the extract towards the pharmacological target relative to dihydrobrevetoxin-B (brevetoxin-3 or PbTx-3). Depending on the sample type, the detection limit (dl) ranged from 5–20 ng PbTx-3 equiv./g or /mL.

#### 2.5.3 Brevetoxin analysis by enzyme-linked immunosorbent assay (ELISA)

The brevetoxin ELISA determines the presence of brevetoxins and brevetoxin metabolites based on cross-reactivity with an anti-brevetoxin polyclonal antibody (i.e., raised in sheep or goat). Two brevetoxin ELISAs were used within this study [57], [58]. Both are direct competitive ELISAs performed in 96-well plate format where brevetoxin-3 was used as the standard. Depending on the sample type, the dl ranged from 2–8 ng PbTx-3 equiv./g or /mL.

#### 2.5.4 Brevetoxin analysis by radioimmunoassay (RIA)

The brevetoxin RIA determines the presence of brevetoxins and brevetoxin metabolites based on a sheep antiserum prepared against a brevetoxin-2 conjugate [59], [60]. The RIA measures the competition between radiolabeled brevetoxin-3 (^3^H-PbTx-3) and unknown samples for the anti-brevetoxin-2 antiserum. The dl of this assay was ∼5 ng PbTx per g (or mL) sample.

#### 2.5.5 Brevetoxin analysis by liquid chromatography-mass spectrometry (LC-MS/MS)

LC-MS/MS methodologies underwent significant evolution over the course of the three UMEs assessed within this study. In 1999–2000, only one brevetoxin congener, brevetoxin-3, was analyzed. By 2004, the availability of additional standards and knowledge of structural information enabled the analysis of multiple metabolites.

For 1999–2000 samples, liquid chromatography was performed on a C18 column using an Agilent Technologies Model 1100 liquid chromatography (LC) system. Five µl of sample extract was separated using a gradient of 1–95% methanol in 0.1% trifluoroacetic acid at a flow rate of 0.2 mL/min. The eluent was introduced into an Applied Biosystems SCIEX API III triple quadrupole mass spectrometer, with an Atmospheric Pressure Chemical Ionization source operated in positive ion mode and nitrogen serving as the nebulization gas. The detection of brevetoxin-3 by mass spectrometry was achieved by multiple reaction monitoring (MRM) with a parent ion of *m/z* 897. Fragment ions characteristic of brevetoxin-3 were obtained at 725, 752, 769 and 807 Daltons. Quantification of brevetoxin-3 was carried out using the 725 Dalton fragment ion by comparison with a standard curve of brevetoxin-3 dilutions (0.1 ng/mL − 10 µg/mL).

For 2004 and 2005/2006 samples, liquid chromatography (LC) separations were performed on a Luna C8(2) 150×2 mm column using an Agilent Technologies Model 1100 LC system followed by mass spectrometry using an AB SCIEX 4000 QTRAP hybrid quadrupole/linear ion trap mass spectrometer equipped with a TurboVTM source interface. The detection and quantification of PbTx congeners by mass spectrometry was achieved by MRM and selected ion monitoring as previously described [19], [61]–[63]. In 2004, only brevetoxin-3 and -9 (tetrahydrobrevetoxin-B) were quantified with a dl of <1.0 ng/mL. In 2005/2006, brevetoxin-3 (B backbone) and brevetoxin-7 (A backbone; dihydrobrevetoxin-A) were quantified. Product ion spectra were used to identify, but not quantify, additional brevetoxin congeners.

#### 2.5.6 Domoic acid extraction

Stomach content and liver samples were extracted by adding four volumes of 50% aqueous methanol and homogenized. The homogenized samples and urine samples were centrifuged at 3000×*g*, and the supernatant passed through a 0.45 µm filter prior to analysis. Blood card samples were extracted for DA using a water (60%)/methanol (40%) solution (2.0 mL). Card extracts were sonicated and extracted for 12 h at 4°C. Extracts were dried under nitrogen gas and resuspended in 100 µL of 10 mM phosphate buffered saline amended with 0.05% Tween All extracts were stored at −20°C until analysis.

#### 2.5.7 Domoic acid analysis by ELISA

A direct competitive DA ELISA (Biosense Laboratories, Norway) [64] was used to screen the samples. This assay measures DA in a sample through its competition with DA coated onto microplate wells for anti-DA antibodies in solution. Extracts were diluted at least 1/20 with ELISA diluent prior to analysis. The dls were variable depending on the sample type and ranged from 0.1 ng/mL up to 60 ng/g.

#### 2.5.8 Domoic acid analysis by LC-MS/MS

Samples or extracts were analyzed for the presence of DA using tandem mass spectrometry coupled with liquid chromatographic separation (LC-MS/MS) using an Agilent 1100 LC coupled to an AB SCIEX API4000 triple quadrupole mass spectrometer in positive ion multiple reaction monitoring mode with methods previously outlined [65], [66]. The DA fragments monitored were *m/z* 266, *m/z* 248, and *m/z* 193. The dl of this method was 1.0 ng DA/mL (urine and blood) and 4.0 ng DA/g (all other samples).

### 2.6 Statistical analysis

All documented *T. truncatus* strandings and *K. brevis* blooms from 1995 to 2006 in the seven Florida Panhandle counties were used to evaluate the temporal relationship between *K. brevis* blooms and *T. truncatus* strandings. Time series cross correlation statistics with month long time lags were used to examine the relationship between monthly bottlenose dolphin strandings and documented *K. brevis* bloom activity each month. To characterize the extent of *K. brevis* bloom in the area of dolphin strandings, the number of documented *K. brevis* reports and the highest *K. brevis* level each month were calculated for the region. Data from the March 2004 event when 105 dolphins stranded with signs of acute intoxication but *K. brevis* was not detected in local waters were excluded from the time series analysis. Statistical analyses were conducted using STATA SE 11.1 (StataCorp, College Station, TX, USA).

## Results and Discussion

### 3.1 Florida Panhandle bottlenose dolphin strandings

**Figure 2:**
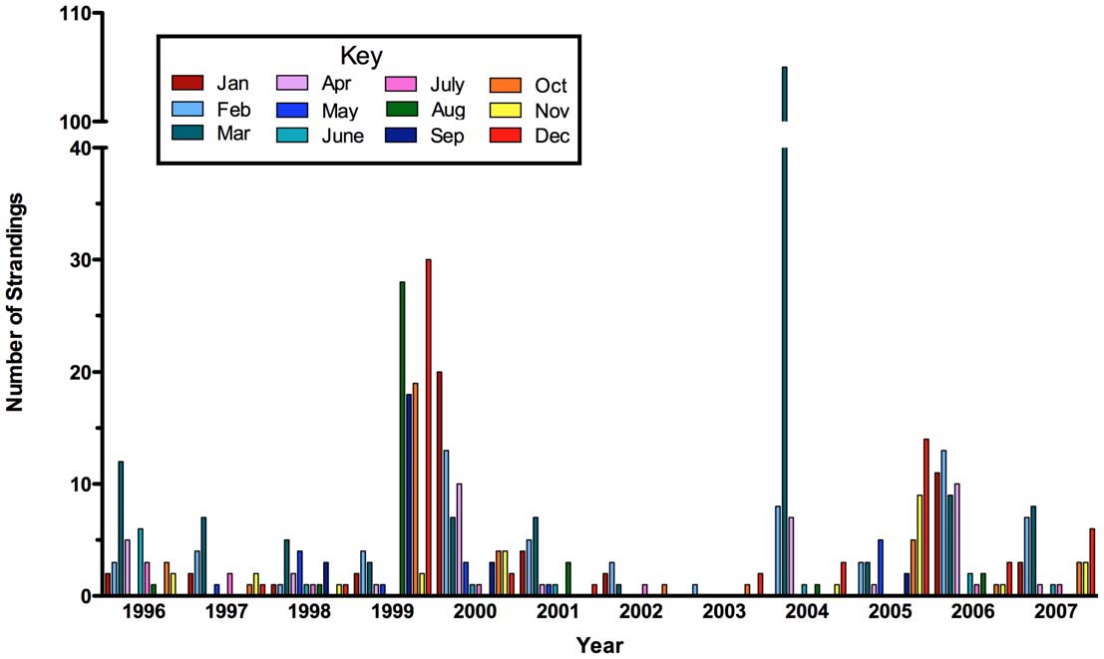
Bottlenose dolphin strandings in the Florida Panhandle region between 1996 and 2007. Note above normal stranding numbers for UMEs during August 1999-May 2000; February-April 2004; September 2005-April 2006).

In the Panhandle region of Florida (USA) (Figure 1) between 1996 and 2007, at least 543 bottlenose dolphins strandings were recorded, averaging 3.8±9.8 strandings per month (Figure 2). Within this region over the 11-year period, three bottlenose dolphin unusual mortality events (UMEs) have been declared by the Office of Protected Resources (NOAA Fisheries); August 1999 to May 2000, February to April 2004, and September 2005 to April 2006. For the 1999/2000 and 2005/2006 events, brevetoxin-producing *K. brevis* was concurrently present (see below), circumstantially suggesting that brevetoxin exposure may have been the cause of death.

### 3.2 1999/2000 Unusual Mortality Event

#### 3.2.1 Harmful algae occurrence

**Figure 3:**
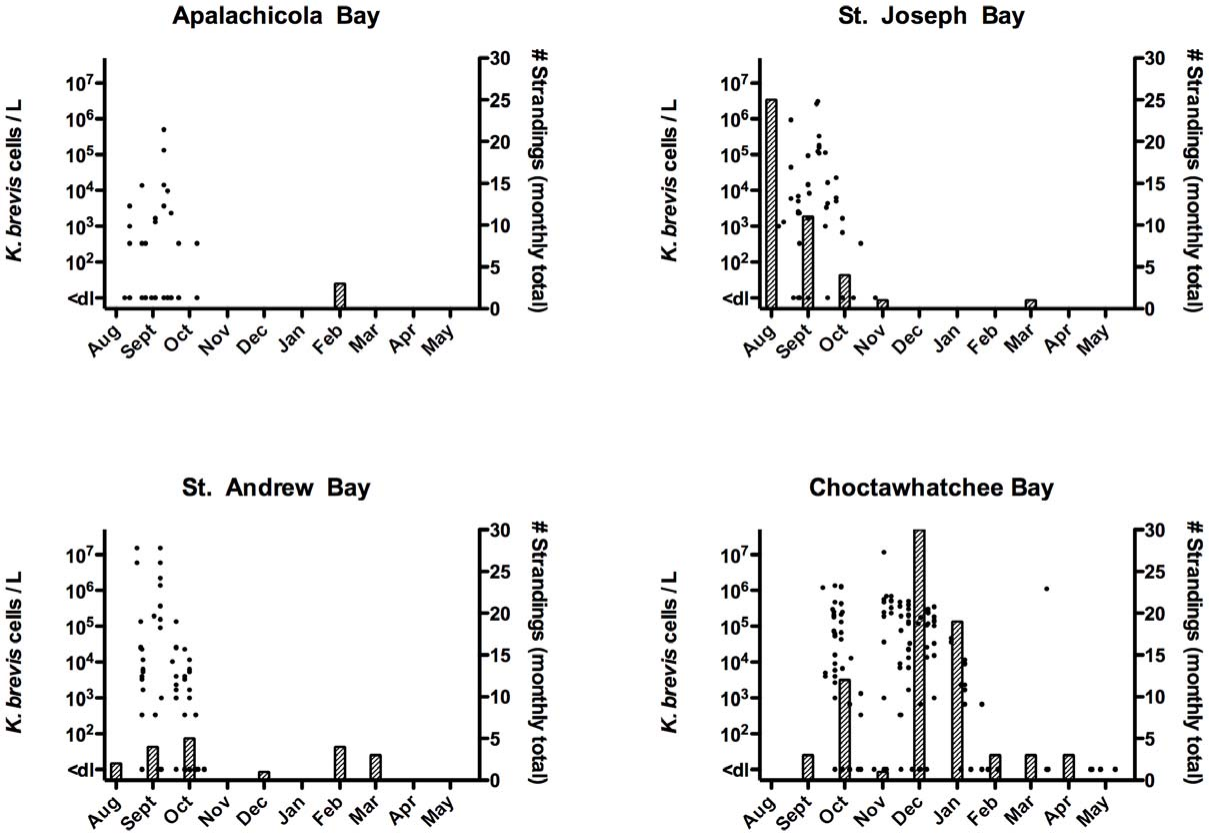
*K. brevis* cell counts and bottlenose dolphin strandings during the 1999/2000 UME in four Bays along the Florida Panhandle. Cell count data (points) are individual measurements from within each Bay at various sites and depths. Stranding data (bars) are compiled based on monthly strandings per county (Apalachicola Bay  =  Franklin County, St. Joseph Bay  =  Gulf County, St. Andrew Bay  =  Bay County, and Choctawhatchee Bay  =  Okaloosa and Walton Counties).

Between August 1999 and January 2000, high densities of *K. brevis* (up to 1.6×10^7^ cells/L) were consistently observed in Panhandle bays (Figure 3) and coastal waters. Reports of fish kills to the FWC Fish Kill Hotline during this UME period reflect the observed bloom conditions. Between August 1999 and December 2000, the FWC Fish Kill Hotline received more than 50 reports of red tide-related fish kills in Panhandle (Franklin to Escambia counties). No reports were received between January and May 2000.

Although the site of bloom initiation is unknown, blooms were initially observed in Apalachicola Bay and St. Joseph Bay before appearing in St. Andrew Bay and then Choctawhatchee Bay from October 1999 until January 2000. This bloom did not enter Pensacola Bay but elevated counts of *K. brevis* (up to 6.0×10^5^ cells/L) were observed in the Pensacola Beach region in late September 1999. Seawater samples collected from St. Joseph Bay in late September 1999 had detectable brevetoxin concentrations in both the intracellular and extracellular fractions [67]. These cell and toxin concentrations are not abnormally high relative to bloom concentrations frequently encountered on the southwest coast of Florida [4], where bottlenose dolphins are exposed to *K. brevis* red tides on an almost annual basis. An ECOHAB-FL cruise in the same near shore waters off Panama City during September 2000 documented similar toxin concentrations within a bloom patch [67], a time period during which there was no apparent increase in bottlenose dolphin mortalities. However, much less information about the extent and breadth of this bloom is available.

#### 3.2.2 Strandings

Over the 40-week 1999/2000 UME period, 152 bottlenose dolphins stranded in two temporally- and spatially-distinct peaks. Peak I from August to September 1999 primarily occurred in St. Joseph Bay whereas Peak II was from December 1999 until January 2000 and primarily occurred in Choctawhatchee Bay (Figure 3). Peak I mortalities began abruptly in St. Joseph Bay with 25 stranded animals in August 1999, 11 in September and 4 in October. Peak II mortalities in Choctawhatchee Bay were well above the historical average in October 1999 (n = 10), low in November (n = 1), followed by mass mortalities in December 1999 (n = 29) and January 2000 (n = 19). Over the course of this event, there were also a significant number of strandings (n = 15) in the Pensacola Bay region. Animals had no overt signs of infectious disease(s) and no consistent gross and histological findings [17]. All stranded bottlenose dolphins tested (n = 68) were of the coastal morphotype according to mitochondrial DNA (P. Rosel, pers. comm.), had a sex ratio of 1∶1, and animals of length 201–239 cm were most common. Although *K. brevis* was continuously present for up to 6 weeks and occasionally exceeded densities of 10^5^ cells/L in both St. Andrew Bay and Apalachicola Bay, very few strandings were observed in these areas (<5 per month). Spatial and temporal trends of bottlenose dolphin strandings and *K. brevis* densities can be viewed in Video S1. No blooms of *Pseudo-nitzschia* were observed in samples collected during this period. Most of the stranded animals were large and robust, but were severely decomposed (code 4 or 5), leaving few (n = 25) animals suitable for algal toxin analysis.

#### 3.2.3 Algal toxin distribution

**Figure 4:**
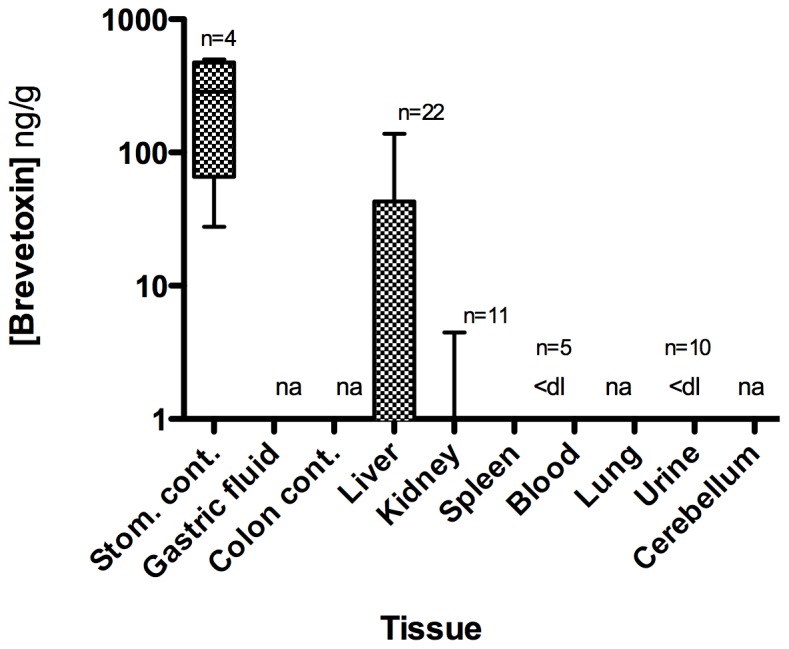
Brevetoxin distribution in various sample types from stranded bottlenose dolphins in the 1999 UME. Values are reported in ng brevetoxin-3 equiv./g. Data are median, quartile, and minimum/maximum values indicated by the midline, box, and whisker lines; respectively. Note: ‘na’ denotes samples were not available for analysis and “<dl” denotes below the detection limit. The number of animals analyzed for each sample type is represented by ‘n = ’.

Brevetoxin was detected in 52% (n = 25) of the animals tested. Liver (n = 23) and kidney (n = 11) composed the majority of samples, following the approach taken in the 1996 Florida manatee mortality investigation [68], with few stomach contents samples being collected. All 4 stomach contents samples analyzed were positive for brevetoxins by RBA and confirmed positive by LC-MS/MS, at concentrations up to >500 ng PbTx-3 equiv./g, strongly suggesting oral exposure. Seven of 22 liver samples and 4 of 11 kidney samples tested positive for brevetoxins, at concentrations up to 138 and 4.4 ng/g, respectively. Brevetoxin was not detected in spleen (n = 5) or lung (n = 10) samples. The distribution of brevetoxin in the various sample types (Figure 4) is similar to the tissue distribution seen in rats following an oral dose of brevetoxin [69] and in other brevetoxin-related marine mammal mortalities [20], [70]. The observation that brevetoxin was highest in stomach and liver suggests that the primary route of exposure in bottlenose dolphins was by ingestion of contaminated prey.

**Figure 5:**
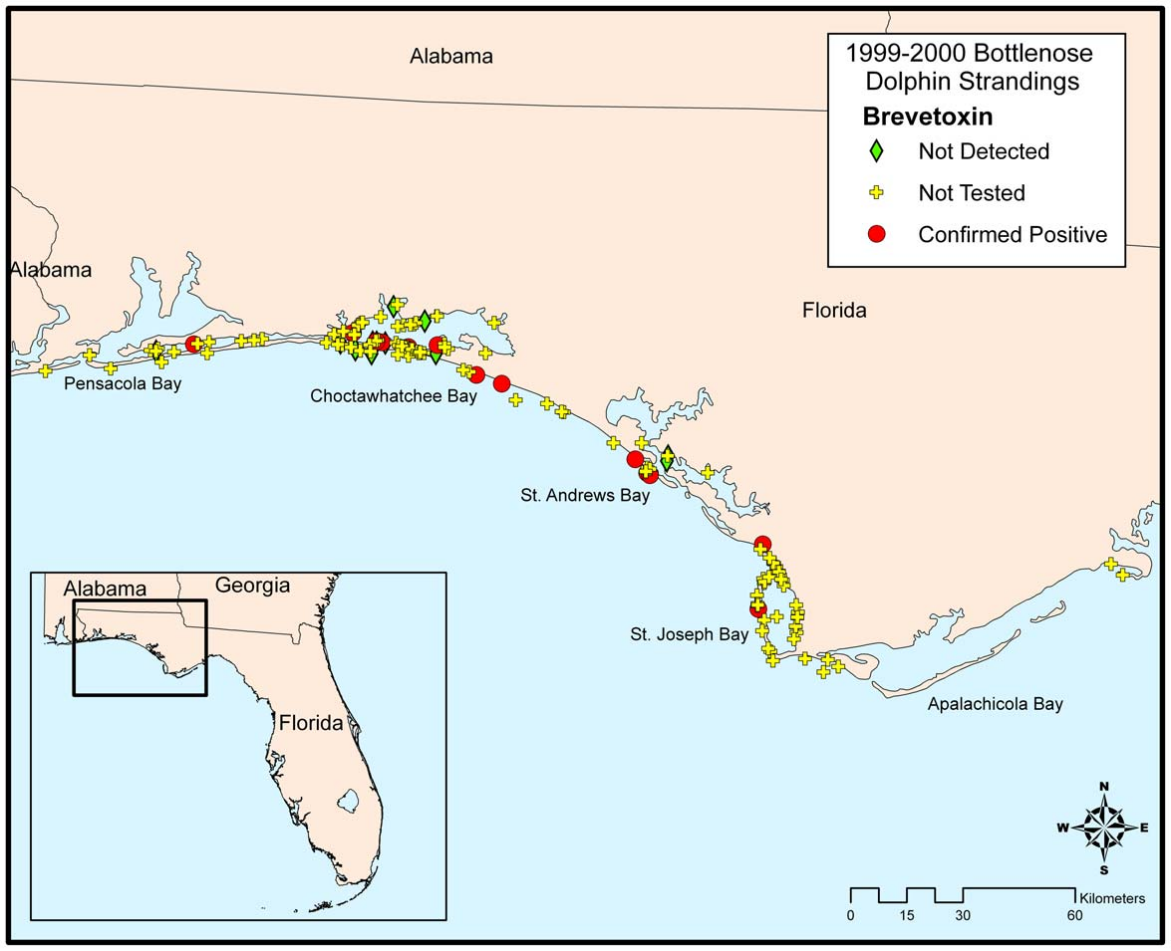
Geographic distribution of 1999/2000 UME bottlenose dolphins that tested positive for brevetoxin.

Although limiting in total sample number, there appears to be some temporal trends of brevetoxin concentrations. Stomach contents positive for brevetoxin were from animals stranding in September 1999 and early January 2000 whereas positive liver samples were observed throughout the event (Table S1). Spatially, animals positive for brevetoxin stranded throughout most of the Panhandle coastline with the greatest number of animals available for toxin analysis stranding in the Choctawhatchee Bay area (n = 11; Figure 5). Of these animals, 5 tested positive for brevetoxin (45%). Although a higher proportion of animals were positive for brevetoxin in St. Joseph Bay (100%), this only constituted 2 animals. Out of the 15 animals that stranded in the Pensacola region, one was shown to contain brevetoxin (Figure 5). Although limited by the lack of sample number, no distinguishable trends were observed in toxin distributions between males and females and length-determined age class (data not shown).

The LC-MS analyses performed at the time were limited to just brevetoxin-3 and its backbone analogs. To investigate for the presence of other congeners and/or metabolites, liver and stomach extracts were fractionated by HPLC and column fractions were collected and tested for cross-reactivity with an anti-brevetoxin antibody using the method of Poli et al. [71]. Fractionation and analysis were performed by Dr. Mark Poli (U.S. Army Medical Research Institute for Infectious Diseases). Brevetoxin-3 was predominant in the 4 stomach contents tested, which were made up primarily of partially digested fish. The presence of brevetoxin-3 in the stomach samples supports the hypothesis that these animals acquired toxin through oral exposure since brevetoxin-3 is the reduced form of brevetoxin-2 that is the predominant toxin analog produced by *K. brevis* [50]. Ten of the 12 brevetoxin-positive livers examined by RIA contained only brevetoxin-3, while two samples had a peak that co-eluted with brevetoxin-2 (data not shown). The identity of this peak was not confirmed due to method limitations at the time, however, it may have represented a metabolite rather than brevetoxin-2, given its known rapid metabolism in other organisms [72].

#### 3.2.4 Algal toxin trophic transfer

At the time of this event, little was known about the capacity for live fish to vector brevetoxins to higher trophic levels, and no live fish were collected as a part of this UME investigation. However, a NOAA-funded ECOHAB research cruise to investigate (in part) fate and effects of *K. brevis* in the food web was coincidentally conducting surveys in the Panhandle region near the onset of this event. During the cruise in late September 1999, several live and apparently healthy Atlantic thread herring (*Opisthonema oglinum*, n = 19) were collected from alongshore of Miramar Beach near Choctawhatchee Bay and analyzed for brevetoxins using a novel detection method (micellar electrokinetic capillary chromatography with laser induced fluorescence detection [73]). *K. brevis* cell densities at the collection site were ranged from 1.2 to 3.0×10^6^ cells/L. Brevetoxins were putatively detected, but not confirmed, at low levels in the stomach and GI tracts (n = 17), in the gills (n = 13), and in the liver (n = 4) [74]. No toxin was detected in the muscle of any of the thread herring.

### 3.3 2004 Unusual Mortality Event

#### 3.3.1 Harmful algae occurrence

**Figure 6:**
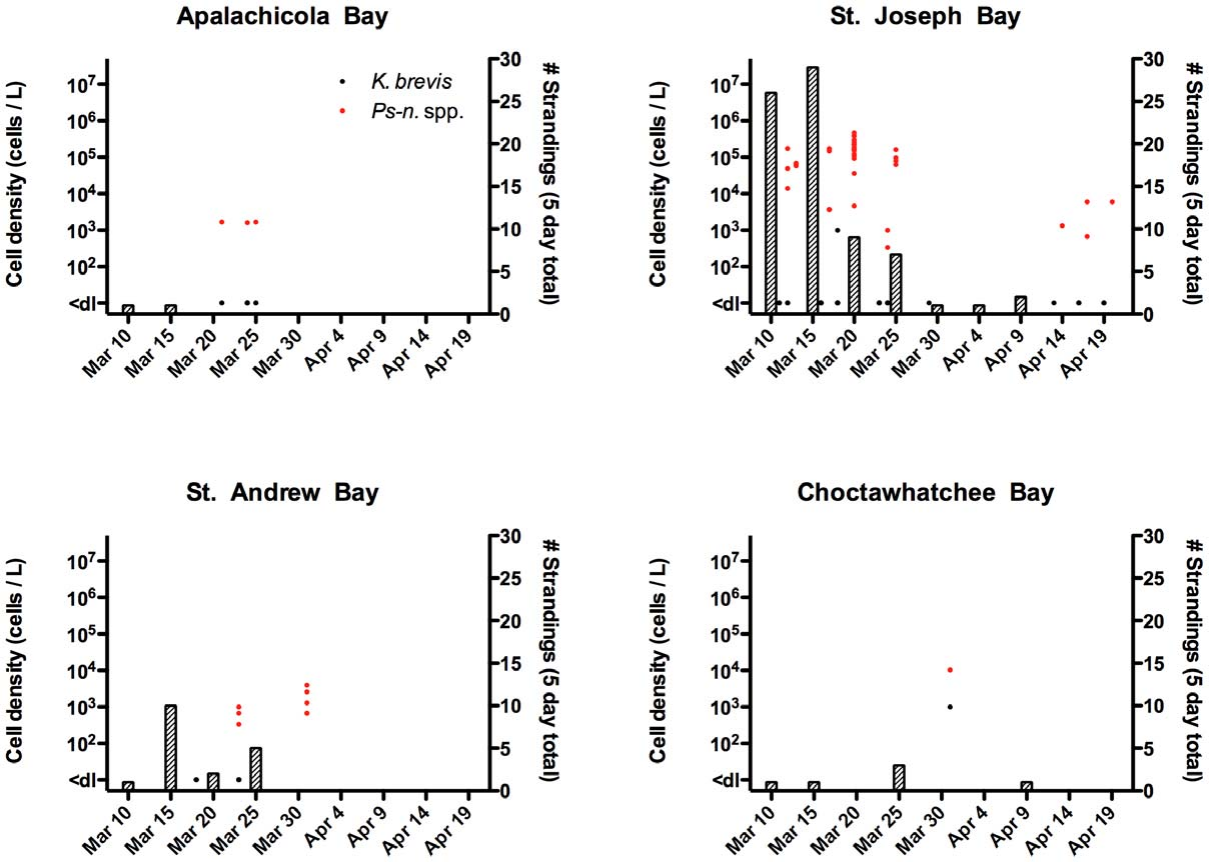
*Karenia brevis* and *Pseudo-nitzschia* spp. cell counts and bottlenose dolphin strandings during the 2004 UME in four Bays along the Florida Panhandle. Cell count data (points) are individual measurements from within each Bay at various sites and depths. Stranding data (bars) are compiled based on 5 day total strandings per county (Apalachicola Bay  =  Franklin County, St. Joseph Bay  =  Gulf County, St. Andrew Bay  =  Bay County, and Choctawhatchee Bay  =  Okaloosa and Walton Counties).

Satellite imagery of the northern Gulf of Mexico indicated elevated chlorophyll levels in the UME area from March 9 to 11, 2004, however, nearly all attempts to document *K. brevis* in the region were unsuccessful (Figure 6). Out of 235 total surface and sub-surface water samples collected only 2 samples were recorded as having low concentrations of *K. brevis* (1000 cells/L). These samples were from St. Joseph Bay and Choctawhatchee Bay on March 18 and March 31, respectively. Out of 82 near-shore and off-shore samples tested for brevetoxin, only 12 samples tested positive but at very low concentrations (<2 μg/L; data not shown). Coincidently, 9 of these samples were from St. Joseph Bay and suggest that a bloom may have been present before, possibly subsurface, in this bay and was just not observed. In contrast, *Pseudo-nitzschia* spp. cell counts in St. Joseph Bay were high between March 12 and March 25 with densities exceeding 10^5^ cells/L in most samples (Figure 6). In the other three bays, *Pseudo-nitzschia* spp. cells were detected but at much lower densities. Unfortunately, *Pseudo-nitzschia* identifications were not made to species level and water samples for DA analysis were all negative (n = 14; unpublished data).

#### 3.3.2 Strandings

Over a four week period starting March 10 and ending April 13, 2004 at least 105 bottlenose dolphins stranded in the Panhandle region with the majority of strandings occurring in St. Joseph Bay (70%, n = 71) and the proximate St. Andrew Bay (17%, n = 18) areas over the first 15 days (Figure 6). Specifically between March 10 and March 19, 55 bottlenose dolphins stranded in St. Joseph Bay resulting in a rapid response by the stranding network. Due to this rapid and massive response, the animal samples that were collected for analysis were of fair condition or better (code 2, 3, or 4) and no overt signs of the cause of death were observed. Stranded bottlenose dolphins had good nutritional status with no evidence of infectious disease(s) (i.e., morbillivirus negative) and no consistent gross and histological findings (pers. comm., D. Rotstein) [54]. All stranded bottlenose dolphins tested (65) were of the coastal morphotype according to mitochrondrial DNA (P. Rosel, pers. comm.), had a sex ratio of 1∶1, and were mostly >240 cm in length. Stranding and cell count data for *K. brevis* and *Pseudo-nitzschia* spp. are spatially and temporally represented in Videos S2 and S3, respectively.

#### 3.3.3 Algal toxin distribution

**Figure 7:**
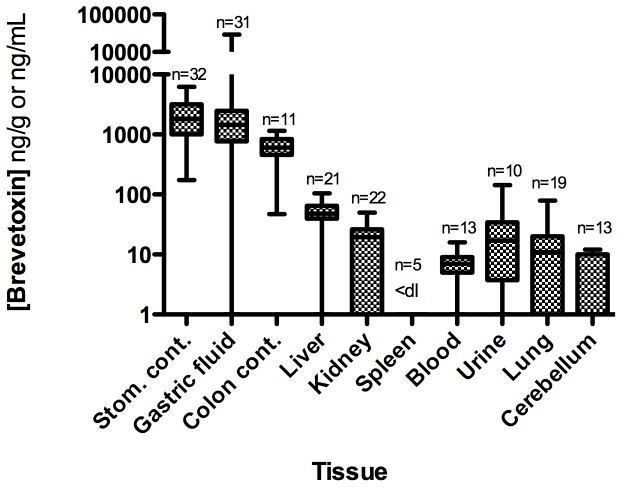
Brevetoxin concentrations in various sample types from stranded bottlenose dolphins in the 2004 UME. Values (mean calculations of all available assay data) are reported in ng brevetoxin-3 equiv./g or ng/mL. Data are median, quartile, and minimum/maximum values indicated by the midline, box, and whisker lines; respectively. Note: “<dl” denotes below the detection limit. The number of animals analyzed for each sample type is represented by ‘n = ’.

Samples from 39 of the 107 bottlenose dolphins that stranded were collected for algal toxin analysis. All bottlenose dolphins tested had very high levels of brevetoxin in their stomach contents with mean ranges between 174 and 6235 ng PbTx-3 equiv./g (Figure 7; Table S2). The stomachs were unusually full of fish prey (mean stomach content weight 773 g) with 10 of 39 samples weighing >1000 g (maximum: 2258 g) [54] suggesting the bottlenose dolphins had just previously fed on and started digesting fish. In many cases, fish from stomach contents were identifiable and available for toxin analysis. Two stomach content samples from animals stranding March 10 and March 11, 2004 were tested using the NSP regulatory mouse bioassay [75] and found to be acutely toxic to mice at levels considered unsafe for humans (30–61 mouse units/100 g). Mean brevetoxin concentrations in gastric fluid ranged from <dl to 29126 ng PbTx-3 equiv./mL, feces (colon contents) from 47 to 1153 ng PbTx-3 equiv./g, and liver from <dl to 104 ng PbTx-3 equiv./g. Urine samples (n = 10) ranged from <dl to 143 ng/mL. Two of these urine samples were tested by LC/MS and confirmed positive for brevetoxin. Studies of brevetoxin metabolism in rats have shown that much of the toxin in urine to be in the form of cysteine-PbTx2 or the oxidized A-ring [76]. Brevetoxin was also identified in all other sample types (kidney, blood, lung, cerebellum) except spleen (as determined by the RBA; Figure 7). The mean order of tissue brevetoxin concentrations (ng/g or ng/mL) were: gastric fluid (2697.3) ∼ stomach contents (2256.9) >> feces (633.0) >> liver (47.9) > urine (29.1) > kidney (18.1) > lung (16.8) > blood (7.2) > cerebellum (4.7) > spleen (<dl).

**Figure 8:**
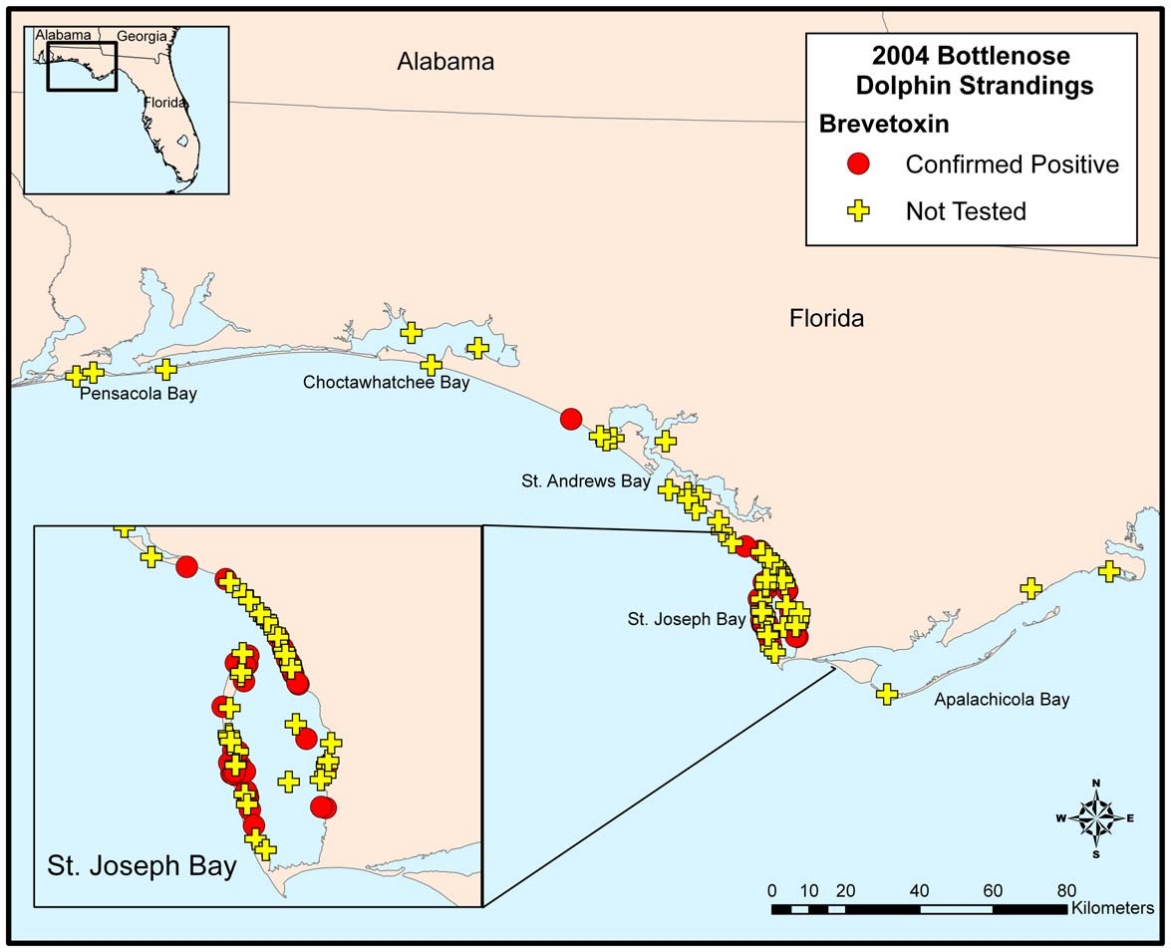
Geographic distribution of 2004 UME bottlenose dolphins that tested positive for brevetoxin.

All animals tested (n = 39) for brevetoxin were positive with all but one (coastal west of St. Andrew Bay) of these animals stranding in St. Joseph Bay (Figure 8). The temporal variability in brevetoxin distribution appears to be unremarkable over the short duration of this event (Table S2) with toxin concentrations in each sample type remaining relatively constant in stranded animals from March 10 until March 27. In fact, of the primary samples analyzed and illustrated in Figure 7 (blood, feces, gastric fluid, liver, stomach contents, urine), only 6 samples were <dl for brevetoxin. Similarly, distinguishable differences were not observed in brevetoxin sample concentrations related to animal sex and age-class over time (data not shown).

Samples from nine bottlenose dolphins were also tested for DA (Table 1). In total, 89% (8 out of 9) of the tested animals were positive for DA. Although all tested liver, kidney, lung, and muscle samples were negative for DA (n = 20), the majority of blood, urine and stomach content samples (87%; 13 out of 15) were positive for low concentrations of DA (<10 ng/g or mL). This is not the first observation of marine mammal exposure to DA in the Gulf of Mexico as DA has been observed in live bottlenose dolphins in Sarasota Bay [40] and in St. Joseph Bay [65]. Although in some cases DA correlated with eosinophilia and putative immunomodulation [65], it is important to note that these levels of DA are 1–2 orders of magnitude lower than the DA concentrations found in lethally poisoned California sea lions on the west coast [26]. Initially it was hypothesized that this finding may help explain the enhanced sensitivity of Panhandle animals to brevetoxin versus Central West Florida animals, however data obtained shortly after the 2004 event using acute, single dose exposures did not suggest any synergistic effects between brevetoxin and DA on mouse lethality (data not shown) or on the cytotoxicity of neuroblastoma cells (Y. Bottein, pers. comm.).

**Table 1:** Domoic acid (DA) concentrations in various samples from stranded bottlenose dolphins in the 2004 UME.

Animal ID	Blood	Urine	Stomach contents	Liver	Kidney	Lung	Muscle
Hubbs-0422-Tt	0.3	2	3	<dl	<dl	<dl	<dl
Hubbs-0423-Tt	0.6			<dl	<dl	<dl	<dl
Hubbs-0428-Tt			8				
Hubbs-0429-Tt	<dl		<dl	<dl	<dl	<dl	
Hubbs-0430-Tt		0.5					
Hubbs-0431-Tt	0.3		7	<dl	<dl	<dl	
Hubbs-0433-Tt	0.3		<dl	<dl	<dl	<dl	
Hubbs-0436-Tt			2				
Hubbs-0437-Tt	0.1		9	<dl	<dl	<dl	

Values (as determined using ELISA) are reported in ng DA/g or ng/mL.

#### 3.3.4 Algal toxin trophic transfer

Stomach content prey items were dominated by Clupeidae and Sciaenidae [54]. Menhaden (*Brevoortia* sp.) was dominant in 14 of 28 (50%) animals examined, sciaenids (i.e., spot, silver perch, kingfish) were dominant in 11 (39%), and mixed taxa including shrimp in the remainder. Several intact or only slightly-digested prey items retrieved from the stomach contents of 19 bottlenose dolphins were analyzed for algal toxins that included fish identified as menhaden (*Brevoortia* sp.), silver perch (*Bairdiella chrysoura*), spot (*Leiostomus xanthurus*), pinfish (*Lagodon rhomboides*), kingfish (*Menticirrhus* sp.), puffer (species unknown), and unidentified teleosts [54]. In many cases the whole fish was homogenized and extracted. All prey items contained high concentrations of brevetoxins with the highest levels measured in the menhaden (Table 2). Whole menhaden (n = 11) ranged from 844 to 15,361 ng PbTx-3 equiv./g and averaged 3,903 ng PbTx-3 equiv./g. Menhaden viscera analyzed separately (n = 3) contained an average concentration of 23,351 ng PbTx-3 equiv./g, and brevetoxins in menhaden muscle averaged 424 ng PbTx-3 equiv./g (n = 6). The importance of menhaden acting as vectors for brevetoxin transfer and for their role in marine mammal brevetoxicosis has been reported previously [20], [77]. Spot and pinfish are also known vectors of brevetoxin into bottlenose dolphins [40] and in this event contained significant levels (181–2048 ng PbTx-3 equiv./g).

**Table 2:** Brevetoxin concentrations in various fish species collected from the stomach contents of 2004 UME bottlenose dolphins.

		Prey item		
Animal ID	Stranding Date	Common Name	Genus species	PbTx-3 equiv. (ng/g)
Hubbs-0415-Tt	3/10/04	Unidentified teleost		2713
Hubbs-0415-Tt	3/10/04	Unidentified teleost		496
Hubbs-0416-Tt	3/11/04	Menhaden	*Brevoortia* sp.	4261
Hubbs-0418-Tt	3/11/04	Menhaden	*Brevoortia* sp.	1442
Hubbs-0418-Tt	3/11/04	Menhaden	*Brevoortia* sp.	4624
Hubbs-0419-Tt	3/11/04	Menhaden muscle	*Brevoortia* sp.	778
Hubbs-0420-Tt	3/11/04	Menhaden muscle	*Brevoortia* sp.	206
Hubbs-0422-Tt	3/11/04	Menhaden	*Brevoortia* sp.	1059
Hubbs-0422-Tt	3/11/04	Menhaden	*Brevoortia* sp.	2300
Hubbs-0423-Tt	3/11/04	Menhaden muscle	*Brevoortia* sp.	446
Hubbs-0423-Tt	3/11/04	Menhaden viscera	*Brevoortia* sp.	31822
Hubbs-0424-Tt	3/11/04	Menhaden	*Brevoortia* sp.	5327
Hubbs-0424-Tt	3/11/04	Menhaden	*Brevoortia* sp.	844
Hubbs-0425-Tt	3/12/04	Silver perch	*Bairdiella chrysoura*	1092
Hubbs-0426-Tt	3/12/04	Spot	*Leiostomus xanthurus*	2048
Hubbs-0426-Tt	3/12/04	Unidentified teleost		828
Hubbs-0427-Tt	3/12/04	Spot	*Leiostomus xanthurus*	536
Hubbs-0428-Tt	3/12/04	Menhaden	*Brevoortia* sp.	15361
Hubbs-0430-Tt	3/10/04	Silver perch	*Bairdiella chrysoura*	3572
Hubbs-0431-Tt	3/10/04	Menhaden	*Brevoortia* sp.	3812
Hubbs-0431-Tt	3/10/04	Puffer sp. muscle		439
Hubbs-0432-Tt	3/11/04	Menhaden muscle	*Brevoortia* sp.	476
Hubbs-0436-Tt	3/12/04	Menhaden muscle	*Brevoortia* sp.	437
Hubbs-0436-Tt	3/12/04	Menhaden viscera	*Brevoortia* sp.	5046
Hubbs-0437-Tt	3/12/04	Kingfish	*Menticirrhus* sp.	1085
Hubbs-0437-Tt	3/12/04	Unidentified teleost		671
SJP-0317-15	3/17/04	Menhaden	*Brevoortia* sp.	939
SJP-0318-17	3/18/04	Menhaden	*Brevoortia* sp.	2968
SJP-0318-17	3/18/04	Pinfish	*Lagodon rhomboides*	405
SJP0325-32	3/25/04	Menhaden muscle	*Brevoortia* sp.	200
SJP0325-32	3/25/04	Menhaden viscera	*Brevoortia* sp.	33185
SJP0325-32	3/25/04	Spot muscle	*Leiostomus xanthurus*	181
SJP0325-32	3/25/04	Spot viscera	*Leiostomus xanthurus*	555

All analyses were conducted by ELISA and expressed as ng brevetoxin-3 equiv./g sample.

Live fish species (66 fish total) caught in the St. Joseph Bay area between March and May, 2004 were analyzed for brevetoxins. Some of these data were previously reported and similarly found that brevetoxins were detected in all fish collected in March 2004 during the UME (Table 3) [18], [77]. Among the March 2004 fish results reported here (n = 12), highest concentrations were measured in the muscle (414 ng PbTx-3 equiv./g.) and stomach contents (3,955 ng PbTx-3 quiv./g) of a spotted seatrout (*Cynoscion nebulosus*). In general, concentrations were lower in fish collected in April 2004 (n = 50), but high concentrations were measured in the liver (mean  = 2334 ng PbTx-3 equiv./g) and stomach contents (mean  = 228 ng PbTx-3 equiv./g) of sheepshead (*Archosargus probatocephalus*). Similar concentrations were measured in the liver (mean  = 2369 ng PbTx-3 equiv./g) and stomach contents (mean  = 251 ng PbTx-3 equiv./g) of spotted seatrout from early May 2004 (n = 4). An undigested clupeid fish recovered from the stomach of one spotted seatrout contained brevetoxin concentrations of 376 and 9075 ng PbTx-3 equiv./g in the muscle and viscera, respectively. The viscera or whole fish of three species (burrfish, pinfish, and spot) were positive for brevetoxin, containing between 3985 and 7755 ng PbTx-3 equiv./g (Table 3).

**Table 3:** Brevetoxin concentrations in various live fish species captured in the St. Joseph Bay region during the 2004 bottlenose dolphin UME.

Common Name	Genus species	Main Diet	n =	Muscle	Liver	Stomach Contents	Viscera	Whole fish
Scaled sardine	*Harengula jaguana*	Plankton	1	18			<dl – 185	
Sheepshead	*Archosargus probatocephalus*	Benthic invert.	4	<dl – 18	65 – 3709	6 – 613		
Spot	*Leiostomus xanthurus*	Benthic invert.	8	<dl – 35			33 – 133	7755
Gulf kingfish	*Menticirrhus littoralis*	Benthic invert.	2	<dl			<dl – 31	
Northern kingfish	*Menticirrhus saxatilis*	Benthic invert.	7	<dl			35 – 77	
Pinfish	*Lagodon rhomboides*	Omnivore	1				4005	
Spotted seatrout	*Cynoscion nebulosus*	Piscivore	7	<dl – 414	<dl – 4655	<dl – 3955		
Leatherjacket	*Oligoplites saurus*	Piscivore	2	<dl			61 – 109	
Southern flounder	*Paralichthys lethostigma*	Piscivore	1	6			90	
Bluefish	*Pomatomus saltatrix*	Piscivore	10	<dl – 45			<dl – 141	
Spanish mackerel	*Scomberomorus maculatus*	Piscivore	23	<dl – 24				
Burrfish	*Chilomycterus schoepfii*	Carnivore	1				3985	
Gulf flounder	*Paralichthys albigutta*	Carnivore	1				<dl	

Data are expressed as ng brevetoxin/g and all analyses were conducted by ELISA or RBA.

### 3.4 2005/2006 Unusual Mortality Event

#### 3.4.1 Harmful algae occurrence

In early October 2005, high densities of *K. brevis* (up to 1.3×10^6^ cells/L) were first observed in Apalachicola Bay and remained elevated until December. Similarly to the west in St. Andrews Bay, cell densities increased over the month of October before peaking at 3.5×10^6^ cells/L in late October and early November and then rapidly declining in December. Over these same time periods, *K. brevis* was observed in Choctawhatchee Bay but at much lower cell densities (up to 2×10^5^ cells/L). Cells were only briefly observed in St. Joseph Bay but reached as high as 2.5×10^6^ cells/L, with maximum brevetoxin concentrations of >80 µg/L [18]. An intense *Pseudo-nitzschia* diatom bloom (up to ∼5×10^6^ cells/L) followed *K. brevis* in St. Joseph Bay, an area that often experiences large diatom blooms (FWC-FWRI's Harmful Algal Bloom database), and in Choctawhatchee Bay but not in the other two Bay systems. Of the water samples collected for DA analysis in St. Joseph Bay in September and November 2005 and March 2006, *Pseudo-nitzschia* spp. were only present at bloom levels (maximum of 7.3×10^5^ cells/L) and DA at concentrations ranging from <dl to 0.7 µg/L in September 2005 (O Dea et al. in prep.).

#### 3.4.2 Strandings

**Figure 9:**
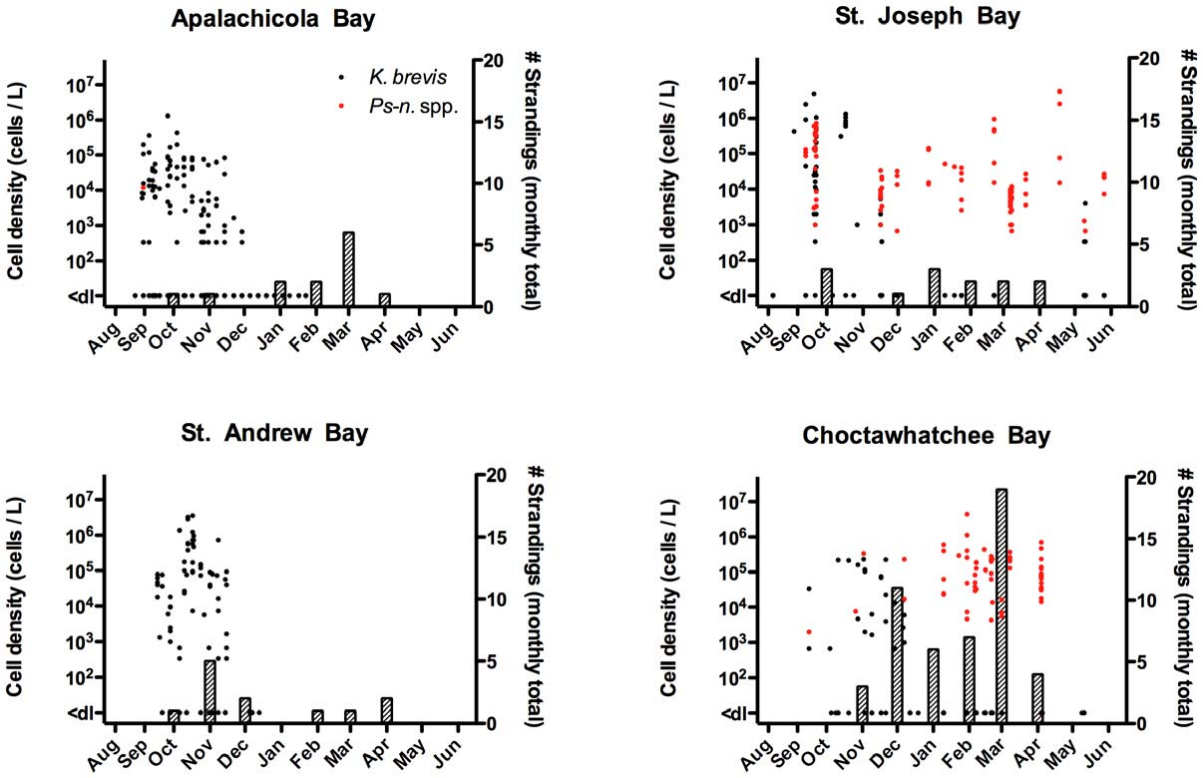
*K. brevis* and *Pseudo-nitzschia* spp. cell counts and bottlenose dolphin strandings during the 2005/2006 UME in four Bays along the Florida Panhandle. Cell count data (points) are individual measurements from within each Bay at various sites and depths. Stranding data (bars) are compiled based on monthly strandings per county (Apalachicola Bay  =  Franklin County, St. Joseph Bay  =  Gulf County, St. Andrew Bay  =  Bay County, and Choctawhatchee Bay  =  Okaloosa and Walton Counties).

Over the 29-week UME event between September 19, 2005 and April 30, 2006, 90 bottlenose dolphins stranded in the Panhandle region with the majority (56%) of these stranding occurring in Choctawhatchee Bay from November 2005 until April 2006 (Figure 9). While strandings were clearly elevated above historical levels from December 2005 to April 2006, there appears to be two peaks of stranded animals; the first peak during the presence of *K. brevis* and the second peak several months following the disappearance of *K. brevis* cells. Maximum strandings in St. Andrew Bay (n = 5) occurred during the bloom whereas maximum strandings in Apalachicola Bay (n = 6) occurred in March 2006, several months after the apparent bloom decline. These animals were generally of fresh to poor condition (code 2, 3, or 4) with some decomposition and no overt signs of the cause of death. They had good nutritional status with no evidence of infectious disease(s) and no consistent gross and histological findings (D. Rostein, pers. comm.). Most stranded bottlenose dolphins tested (62 of 64) were of the coastal morphotype according to mitochondrial DNA (P. Rosel, pers. comm.), had a sex ratio of 1∶1, and were mostly of the 201–240 cm length size class. Bottlenose dolphin stranding and algal cell count data for *K. brevis* and *Pseudo-nitzschia* spp. are spatially and temporally represented in Videos S4 and S5, respectively.

#### 3.4.3 Algal toxin distribution

**Figure 10:**
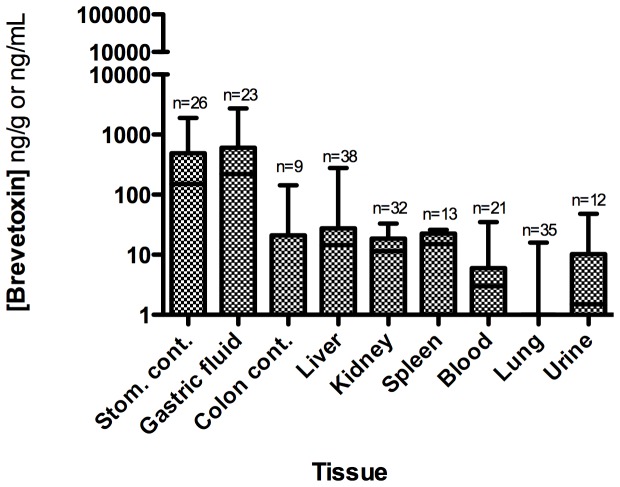
Brevetoxin concentrations in various sample types from stranded bottlenose dolphins in the 2005/2006 UME. Values (mean calculations of all available assay data) are reported in ng brevetoxin-3 equiv./g or ng/mL. Data are median, quartile, and minimum/maximum values indicated by the midline, box, and whisker lines; respectively. Note: The number of animals analyzed for each sample type is represented by ‘n = ’.

Samples from 41 of the 89 bottlenose dolphins that stranded were collected for algal toxin analysis (Figure 10). Stomach contents contained brevetoxin levels similar to gastric fluid (<dl to 2724 ng PbTx-3 equiv./mL) and liver samples contained brevetoxin at concentrations (<dl to 278 ng PbTx-3 eq./g) similar to the 1999/2000 UME events (<dl to 138 ng PbTx-3 equiv./g) and 2004 (<dl to 104 ng PbTx-3 equiv./g). Many of the blood samples tested positive for brevetoxin, as did kidney, lung, cerebellum, with the occasional blubber and muscle sample also positive. In contrast to the 2004 data, 5 out of 8 spleen samples from 2005/2006 were positive. However, this discrepancy may be a result of the different assays used between these two events, since the ELISA method used to analyze spleen samples in 2005/2006 is more sensitive than the RBA method used in 2004. Unlike the acute 2004 UME, many of the feces and urine samples from 2005/2006 tested negative suggesting different toxin elimination kinetics and likely exposure scenarios between these events. The mean order of tissue brevetoxin concentrations (ng/g or ng/mL) were: gastric fluid (508.6) ∼ stomach contents (351.6) >> liver (31.0) ∼ feces (21.7) > spleen (13.4) ∼ kidney (10.8) ∼ urine (7.6) ∼ blood (5.2) > lung (1.8).

An interesting occurrence within this event was the recovery of 2 sets of mother – fetal pairs for algal toxin analysis that appear to represent unique scenarios. Data gathered via a rodent model has shown that pregnant mothers exposed to brevetoxin-3 can transfer this toxin to their fetuses via placental transport [78]. In PCNMFS-06-07 mother, all samples (including amniotic fluid) were negative for brevetoxin with the exception of 9 ng PbTx-3 equiv./g in the liver (Table S3) suggesting a previous low dose or protracted exposure. Nonetheless, the stomach contents and liver collected from the fetus of PCNMFS-06-07 were negative for brevetoxin. The female FLCB030206-11 had at least one brevetoxin-positive shrimp in the stomach (780 ng PbTx-3 equiv./g), brevetoxin in the blood, liver, kidney, spleen, and amniotic fluid, but not in the gastric fluid, lungs, or muscle. However, no brevetoxin was found in the liver or feces of the fetus. Collectively, these data suggest that maternal transfer may be possible but firm conclusions cannot be made due to limitations in sample size.

**Figure 11:**
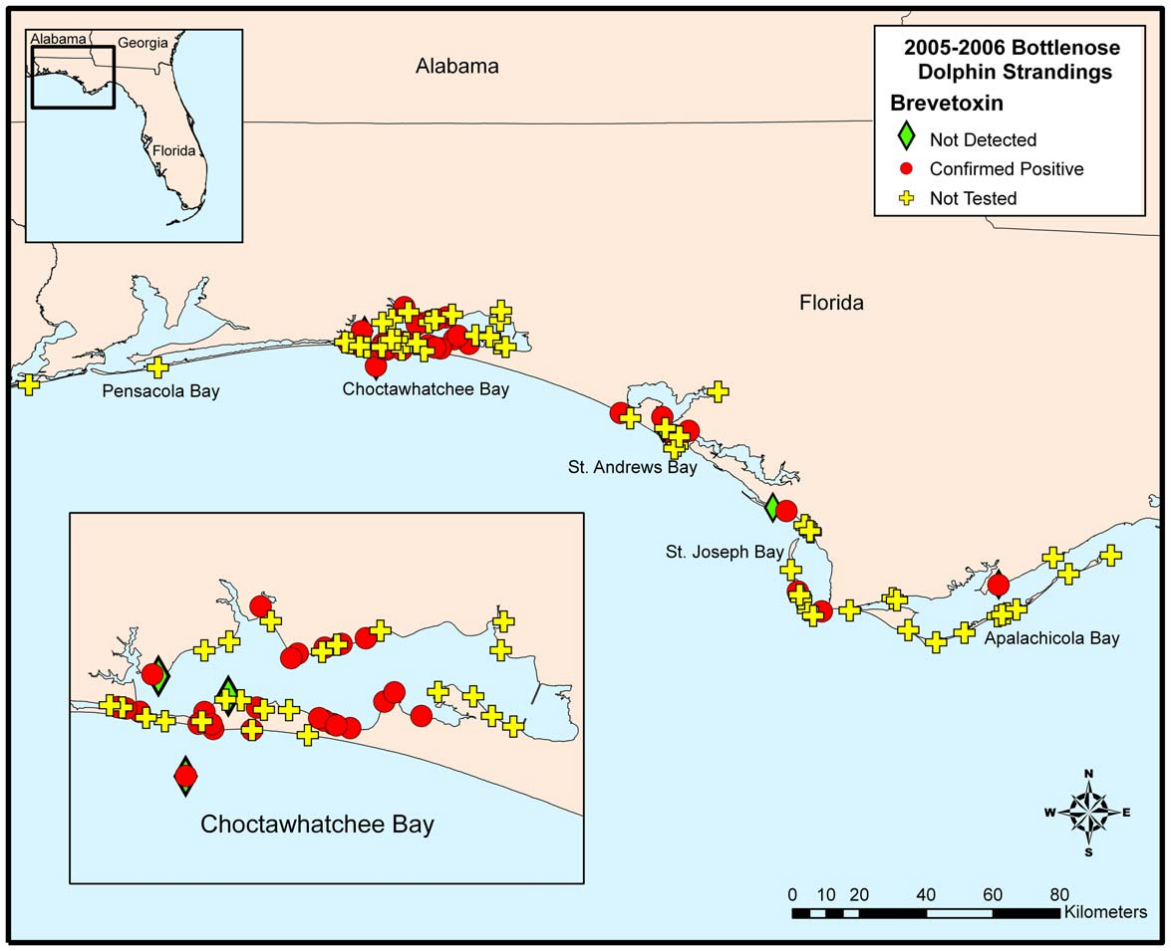
Geographic distribution of 2005/2006 UME bottlenose dolphins that tested positive for brevetoxin.

Thirty-eight of the 41 tested animals (93%) were positive for brevetoxin with the majority of strandings occurring in Choctawhatchee Bay (Figure 11). Temporal variability in brevetoxin tissue/sample concentrations appears to be unremarkable over the duration of this event (Table S3) with toxin concentrations in each sample type remaining relatively constant in stranded animals from November 2005 until April 2006. Similarly, distinguishable differences were not observed in brevetoxin sample distributions related to animal sex and age-class over time (data not shown). However, a disproportionate number of dolphins with length ranges of 200–240 cm were analyzed relative to the higher number of dolphins in this length range that stranded.

After measuring low levels of DA in some 2004 UME bottlenose dolphin samples (<dl to 9 ng/g or ng/mL; Table 1), DA was assessed in bottlenose dolphin samples collected during the 2005/2006 event. Using ELISA, liver (n = 5), stomach contents (n = 5), and urine (n = 9) all tested negative for DA (data not shown). This absence of DA was not due to the absence of *Pseudo-nitzschia* spp. cells in the area since from December 2005 until April/May 2006 cell counts in St. Joseph Bay and Choctawhatchee Bay remained high and at times exceeded 10^6^ cells/L (Figure 9). Furthermore, in September 2005 DA was identified in the area (O Dea et al. in prep.).

#### 3.4.4 Algal toxin trophic transfer

Most animals (20 of 27) had at least one food item in their stomachs that tested positive for brevetoxin (range <dl to 2034 ng PbTx-3 equiv./g; Table 4), however the concentrations tended to be several fold lower than concentrations observed during the 2004 UME (range 401 to 10080 ng PbTx-3 equiv./g) (Table 2) [20] but greater than the 1999 UME event (range 28 to 393 ng PbTx-3 equiv./g). Although limited in number due to advanced stages of digestion, stomach content analysis identified many small fish species including menhaden and spot that contained brevetoxin. In particular, spot have been found in greater abundance in dolphin stomachs during and shortly after blooms of *K. brevis* compared to dolphin stomach samples collected in the absence of bloom activity [79]. For the first time, we also observed an abundance of shrimp in the stomach contents of these animals. Although highly variable, many of these shrimp also contained brevetoxin (<dl to 760 ng PbTx-3 equiv./g; Table 4), possibly representing an additional undocumented trophic route distinct from planktivorous fish. We found a high prevalence of brevetoxins in live fish collected during and immediately after the *K. brevis* bloom, with maximum concentrations reaching 414 ng PbTx-3 equiv./g in muscle and 4655 ng PbTx-3 equiv./g in liver (Table 3). In a 2-year follow-up study focused on St. Joseph Bay by Naar et al. [18], brevetoxins continued to be present in the livers of live fish for more than a year after the cessation of the bloom. A multispecies persistent fish kill in Choctawhatchee Bay from mid-January to May 2006, with juvenile spot being the most affected, correlated with high tissue concentrations of brevetoxins (up to 6438 ng PbTx-3 equiv./g in intestinal content), even in the apparent absence of *K. brevis* cells [21]. These data support the role of brevetoxin trophic transfer in both acute mortalities during *K. brevis* blooms (i.e., Choctawhatchee Bay, December 2005), and the delayed or protracted impacts on bottlenose dolphins many months following a *K. brevis* bloom due to complex retention and distribution throughout the food web (i.e., Choctawhatchee Bay, March 2006) potentially contributing to spatial and temporal lags in animal exposures to toxins. Alternatively, the March 2006 elevation in strandings could have been the result of a *K. brevis* bloom that went undetected; not unlike what is speculated for the 2004 UME.

**Table 4:** Brevetoxin concentrations in various fish species collected from the stomach contents of 2005/2006 UME bottlenose dolphins.

		Prey item		
Animal ID	Stranding Date	Common Name	Genus species	PbTx-3 equiv. (ng/g)
PCNMFS-05-05	11/1/05	Unidentified teleost		966
PCNMFS-05-13	12/13/05	Spot	*Leiostomus xanthurus*	494
PCNMFS-05-18	12/19/05	Menhaden	*Brevoortia* sp.	1511
PCNMFS-05-19	12/26/05	Unidentified teleost		1543
PCNMFS-06-06	1/13/06	Shrimp	*Penaeus* sp.	<dl
PCNMFS-06-06	1/13/06	Unidentified teleost		2034
PCNMFS-06-06	1/13/06	Unidentified teleost		<dl
FLCB022806-09	2/28/06	Shrimp	*Penaeus* sp.	<dl
FLCB030206-10	3/1/06	Shrimp	*Penaeus* sp.	583
FLCB030206-11	3/2/06	Shrimp	*Penaeus* sp.	760
FLCB031406-17	3/14/06	Unidentified teleost		692
FLCB031606-19	3/16/06	Unidentified teleost		<dl
FLCB032406-23	3/24/06	Shrimp	*Penaeus* sp.	188
PCNMFS-06-18	4/1/06	Menhaden	*Brevoortia* sp.	<dl

All analyses were conducted by RBA.

### 3.5 Correlations between *Karenia brevis* and bottlenose dolphin strandings

*K. brevis* was documented in one or more of the seven Florida panhandle counties 1945 times between June 1995 and December 2006, including 55 of the 144 months. During the same time period 567 bottlenose dolphin strandings were documented representing 101 of the 144 months. Dolphin strandings were marginally correlated with the number of *K. brevis* reports each month (Pearson cross correlation coefficient 0.16, P = 0.051). Strandings were significantly positively correlated with the number of *K. brevis* reports each month when the bloom data were time lagged so they preceded strandings by 1, 3, 4 and 5 months. The strongest positive correlations were detected when *K. brevis* bloom data preceded stranding data by 3, 4, and 5 months with cross correlation coefficients 0.272. 0.274, and 0.256, respectively (p<0.05). Bottlenose dolphin strandings were similarly correlated in time when *K. brevis* activity was measured using the highest *K. brevis* count reported for that month. While strandings and bloom level were not significantly correlated in real time (Pearson correlation coefficient 0.143, P = 0.086), highest monthly bloom level was highly correlated with strandings when bloom data preceded strandings by 1 month (Pearson correlation coefficient 0.345, P<0.05). As was noted for the other measure of local *K. brevis* activity, significant correlations were detected when data on highest monthly *K. brevis* count preceded strandings by 3, 4, and 5 months [54].

## Conclusion

### 4.1 Algal toxin-related UMEs in Florida

According to the Marine Mammal Protection Act, the declaration of a UME is based on “a stranding that is unexpected, involves a significant die-off of any marine mammal population, and demands immediate response”. Between 1999 and 2006, there were three UMEs involving the mass mortality of bottlenose dolphins in the Florida Panhandle region. Although cursory circumstantial evidence had suggested the involvement of *K. brevis*, substantive toxin data confirming exposure to brevetoxins have not been forthcoming until now. Based on the analysis of 105 stranded animals and over 850 analytical or bioassay methods (i.e., ELISA, receptor binding assay, radioimmunoassay, LC-MS/MS) that have confirmed the presence of brevetoxin in 86% of the stranded animals tested, our data provide the first link between the deaths of Panhandle bottlenose dolphins and *K. brevis* and/or brevetoxins in each of the 1999/2000, 2004, and 2005/2006 mortality events.

An interesting discrepancy in the Gulf of Mexico is that documented *K. brevis* cell counts in the Panhandle region are no higher (and in many cases lower) than those typically and frequently observed during blooms along the Central West Florida region [4] with known exposures to local bottlenose dolphin populations [61], [80] (Table 5), but yet the impacts on bottlenose dolphins and their contribution to mass mortalities are vastly different. Over the 1999–2006 study period, there were two known *K. brevis* bloom events (1999/2000 and 2005/2006) in the Panhandle region and each of these events coincided with major mortality events that were declared UMEs. However, during the same period in Central West Florida, there were major blooms nearly every year but only one UME bottlenose dolphin mortality event in 2005/2006 (Table 5). This difference in susceptibility could be due to the observation that Panhandle blooms have a tendency to become trapped in embayments such as St. Joseph Bay, thereby potentially increasing brevetoxin exposure periods and/or potentially higher accumulation in the prey base of the local trophic system. In addition, this study and a study involving live bottlenose dolphins also highlight the presence of another algal toxin, domoic acid (DA), that is present in Panhandle bottlenose dolphins [65]. However, the levels of DA were very low when compared to stranded marine mammals on the west coast of North America [26] and DA has just recently been documented in live Sarasota Bay bottlenose dolphins that reside in the Central West Florida region [40]. Another explanation for the sensitivity discrepancy between the Pandhandle and Central West Florida dolphin populations is that since *K. brevis* blooms are nearly annual events in Central West Florida, the resident bottlenose dolphins in these areas have become desensitized to the harmful effects of brevetoxin. Alternatively, the bottlenose dolphins residing in the Panhandle region are much more sensitive to the harmful effects of brevetoxin. This difference in susceptibility between the two regions does not appear to be due to population genetic differences related to morphotype. DNA analysis of the bottlenose dolphins affected by the two concurrent 2005/2006 UMEs determined that both populations were dominated by the coastal morphotype (P. Rosel, pers. comm.). Finally, differences in feeding habits between Panhandle and Central West Florida dolphin populations may be another factor in resolving this discrepancy. Sarasota Bay dolphins generally feed on solitary, seagrass-associated fish that are a mix of herbivores, carnivores, detritivores and omnivores [19], [41], whereas the 2004 Panhandle UME dolphins were intoxicated by feeding on menhaden, which are offshore, pelagic planktivores [20]. The fact that dolphins from these two regions may be feeding at multiple trophic levels may result in differential brevetoxin exposure that cannot be resolved by simply comparing brevetoxin concentration ranges during UMEs. Despite the regional differences in sensitivity, it is quite clear that in both regions the presence of highly dense *K. brevis* blooms results in a higher percentage of animals exposed to brevetoxin, higher concentrations of brevetoxin in various tissues/fluids, and a greater chance for an UME (Table 5). This is particularly evident when compared to samples obtained during live dolphin health assessments in St. Joseph Bay and Sarasota Bay, FL during periods of low bloom activity (i.e., *K. brevis* cells counts <1000 cells/L) [40], [65].

**Table 5:** Summary of brevetoxin exposure to live and dead Gulf of Mexico bottlenose dolphins.

						[PbTx] range; ng/g or ng/mL						
Region	Location	Year(s)	Mortality event?; # dead	*K. brevis* present; max cells/L (x10^6^)	% of tested animals PbTx positive	GI Contents/fluids	Liver	Feces	Urine	Blood/serum	Comments	Reference
**Northern Gulf of Mexico Dolphins**												
	Northwest Florida	1999–2000	yes; 162	16	52%	28-500+	<dl–138					current study
	Northwest Florida	2004	yes; 107	≤0.001	100%	<dl –29,126	<dl–104	<dl–1,152	<dl–143	<dl–16		current study
	Northwest Florida	2005–2006	yes; 89	4.8	93%	<dl–2,724	<dl–278	<dl–143	<dl–48	<dl–35		current study
	Northwest Florida	2005–2006	no	≤0.001	47%				<dl	<dl–1.1	23%: elevated eosinophils	[65]
	Texas	2008	yes; 119	≤0.001	13%	<dl		<dl–19	<dl			[62]
**Southwest Florida Dolphins**												
	Sarasota, FL	1994, 1999, 2001	no	≤0.001	56%		<dl–21				Strandings during non-*K. brevis* bloom periods	[80]
	Sarasota, FL	2000, 2004, 2006, 2008, 2009	no	≤0.001	36%	<dl–10		<dl–109	<dl–90	<dl	Live captures during non-*K. brevis* bloom periods	[40]
	Sarasota, FL	2001–2003	no	105	84%	<dl–2,896	<dl–71	288 – 749	<dl–100		Strandings during *K. brevis bloom*, no mortality event declared	[80]
	Sarasota, FL	2004, 2005	no	3.8	38%				<dl–7	<dl –2	Live captures during *K. brevis* bloom periods	[40]
	Southwest Florida (multiple counties)	2005–2006	yes; 190*	162	72%	<dl–26,390	<dl–788	<dl–774	<dl–54	<dl–13	Central West Florida Multi-Species 2005–2006 Unusual Mortality Event	NOAA and FWC, unpublished data

* All dolphin strandings in Pinellas, Manatee, Sarasota, Charlotte, and Collier counties during specified time period between July 2005 and Nov 2006, including 17 live strandings and all causes of death, known and unknown.

### 4.2 Contributions to a case definition of brevetoxicosis for bottlenose dolphins

Data from the three distinct UMEs presented in this study will significantly contribute to a brevetoxicosis case definition for bottlenose dolphins, which has been identified as a priority need to better understand the acute and chronic impacts of brevetoxin exposure on individuals and populations of bottlenose dolphins [81]. Criteria may include evidence of brevetoxin exposure via the detection of brevetoxin in the gastrointestinal compartment (i.e., stomach contents, gastric fluid, and/or feces) as well as evidence of brevetoxin uptake via the detection of brevetoxin in internal fluids (i.e., blood, urine) and/or tissues (liver, kidney, etc). However, specific threshold levels will be difficult to ascribe because of the various rates associated with distribution and/or metabolism specific to each compartment/fluid/tissue. As well, carcass condition and age presumably affects toxin levels, particularly degradation. Blooms of brevetoxin-producing *K. brevis* may or may not be present. Additionally, other causes of death should be ruled out, including physical injuries (i.e., ship strikes, natural predators, fishing gear entanglements), age, and exposure to pathogenic microbes (viruses and bacteria) and parasites. Exposure to other contaminants (i.e., organochlorines, metals, etc) should be at non-concerning levels. With these toxicological factors in mind, it seems appropriate that all three events presented in this study (1999/2000, 2004, and 2005/2006 UMEs) would be classified as events of brevetoxicosis.

### 4.3 Factors necessary for brevetoxin-induced UME

Although there are certainly differences between the three UMEs outlined in the current study, there are distinct commonalities that appear to precede a bottlenose dolphin UME in the Gulf of Mexico. First and foremost, whether it is possible to detect it or not, there must be a dense *K. brevis* bloom producing brevetoxin. *K. brevis* cells must then subsequently be consumed by planktivorous organisms such as fish (i.e., menhaden) or benthic organisms such as shrimp. These toxin-containing fish or invertebrates can be directly consumed by bottlenose dolphins or consumed by small piscivorous fish (i.e., seatrout), that biomagnify brevetoxin and can then be consumed by dolphins or alternately retain the toxin for as long as one year following their initial exposure [18]–[20]. The lag time between a *K. brevis* bloom and dolphin mortality is probably related to the degree of trophic transfer and toxin dispersal that occurs between the bloom and dolphin consumption of toxin-containing fish [21] and the feeding preferences of the dolphins. More direct transfer of toxin to dolphins likely results in dolphin mortality and stranding in conjunction with or closely following a bloom of *K. brevis* and was likely the case in St. Joseph Bay in 1999 and 2004 as well as in Choctawhatchee bay in 2005. Although much more difficult to ascertain, evidence within this study and others [19], [40] suggest that there were alternate and more protracted (spatially and temporally) routes of trophic transfer that likely account for the significant time lag between a *K. brevis* bloom and the second peak of the 1999/2000 UME as well as the 2005/2006 dolphin stranding event. This alternate trophic route appears to have involved more demersal (i.e., benthic) fish species such as spot, pinfish, and kingfish that may accumulate brevetoxins from the sediments, seagrass, and/or benthic invertebrates [79].

### 4.4 Future directions

It is generally acknowledged that there is an apparent increase in the incidence and intensity of HABs [3], [82] that potentially is influenced by natural and anthropogenic factors, including eutrophication [83] and global climate change [84]. If this represents an actual increase in the frequency and duration of bloom events such as brevetoxin-producing *K. brevis* and DA-producing *Pseudo-nitzschia* spp., there will likely be a greater chance of concurrent and synergistic exposure of aquatic organisms such as fish and marine mammals to these types of HAB toxins resulting in mass mortality events [55]. It is therefore imperative that future studies attempt to gain a better understanding of the ecological circumstances that result in algal toxin-related marine mammal exposure and mortality.

## Supporting Information

Video S1 **Dolphin strandings and** ***K. brevis*** **cell counts across space and time in the Florida Panhandle region, USA during the 1999/2000 UME event.**
(MP4)

Video S2 **Dolphin strandings and** ***K. brevis*** **cell counts across space and time in the Florida Panhandle region, USA during the 2004 UME event.**
(MP4)

Video S3 **Dolphin strandings and** ***Pseudo-nitzschia*** **spp. cell counts across space and time in the Florida Panhandle region, USA during the 2004 UME event.**
(MP4)

Video S4 **Dolphin strandings and** ***K. brevis*** **cell counts across space and time in the Florida Panhandle region, USA during the 2005/2006 UME event.**
(MP4)

Video S5 **Dolphin strandings and** ***Pseudo-nitzschia*** **spp. cell counts across space and time in the Florida Panhandle region, USA during the 2005/2006 UME event.**
(MP4)

Table S1 **Brevetoxin concentrations in various tissues from stranded dolphins in the 1999/2000 UME.** Values are reported in ng PbTx-3 equiv./g.
(DOCX)

Table S2 **Brevetoxin concentrations in various tissues from stranded dolphins in the 2004 UME.** Values are reported in ng PbTx-3 equiv./g or ng/mL.
(DOCX)

Table S3 **Brevetoxin concentrations in various tissues from stranded dolphins in the 2005/06 UME.** Values are reported in ng PbTx-3 equiv./g or ng/mL.
(DOCX)
