# Supplementary material for: Comparative Analysis of Three Brevetoxin-Associated Bottlenose Dolphin (Tursiops truncatus) Mortality Events in the Florida Panhandle Region (USA)
Source: PLoS One. 2012 Aug 15;7(8):e42974. doi: 10.1371/journal.pone.0042974 (PMC3419745; doi:10.1371/journal.pone.0042974)

**Table S1. Brevetoxin concentrations in various tissues from stranded dolphins in the 1999/2000 UME.** Values are reported in ng PbTx-3 equiv./g.


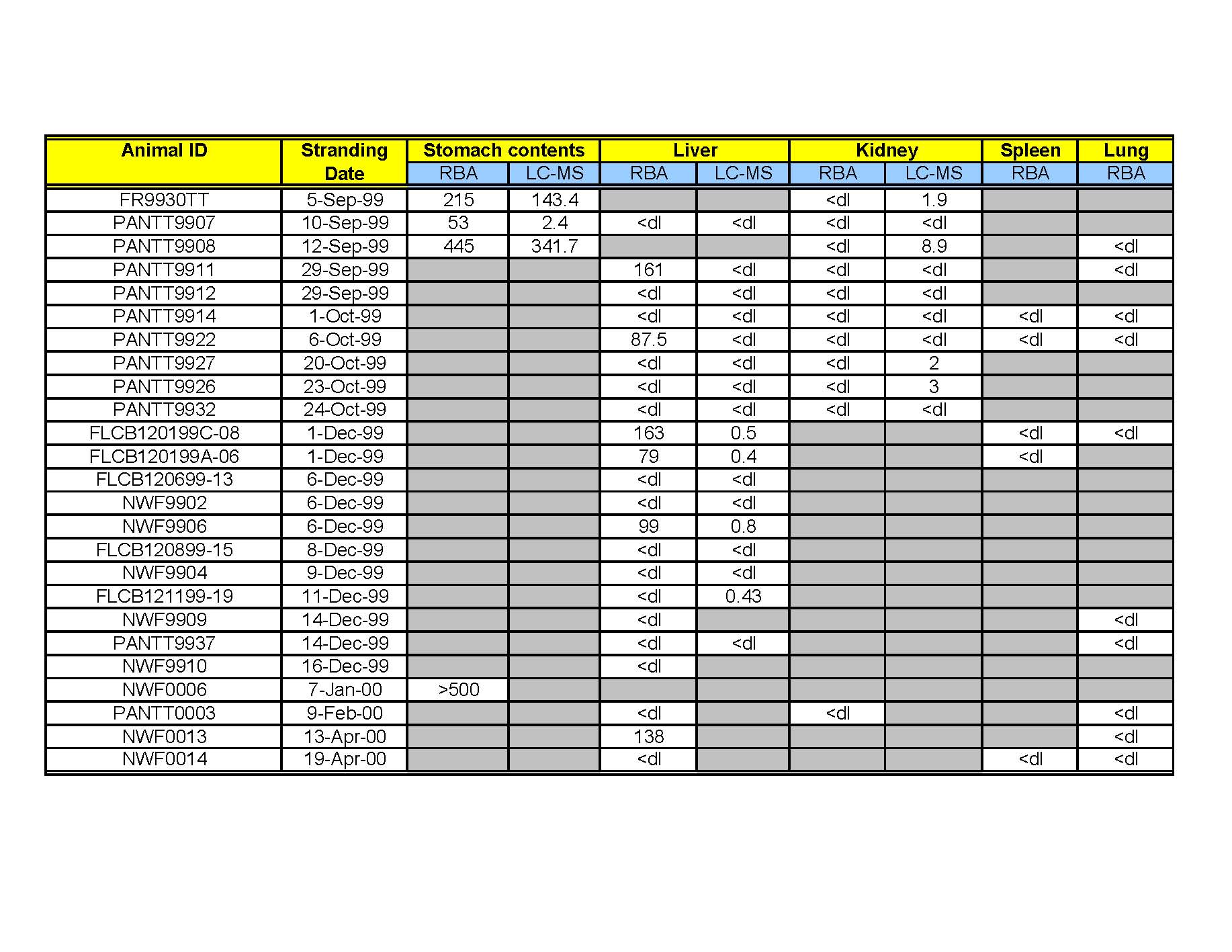

Supplement: Table S1 — Brevetoxin concentrations in various tissues from stranded dolphins in the 1999/2000 UME. Values are reported in ng PbTx-3 equiv./g. (DOCX) [file pone.0042974.s006.docx]
