# Supplementary material for: Comparative Analysis of Three Brevetoxin-Associated Bottlenose Dolphin (Tursiops truncatus) Mortality Events in the Florida Panhandle Region (USA)
Source: PLoS One. 2012 Aug 15;7(8):e42974. doi: 10.1371/journal.pone.0042974 (PMC3419745; doi:10.1371/journal.pone.0042974)
Supplement: Table S2 — Brevetoxin concentrations in various tissues from stranded dolphins in the 2004 UME. Values are reported in ng PbTx-3 equiv./g or ng/mL. (DOCX) [file pone.0042974.s007.docx]

**Table S2. Brevetoxin concentrations in various tissues from stranded dolphins in the 2004 UME.** Values are reported in ng PbTx-3 equiv./g or ng/mL.

*
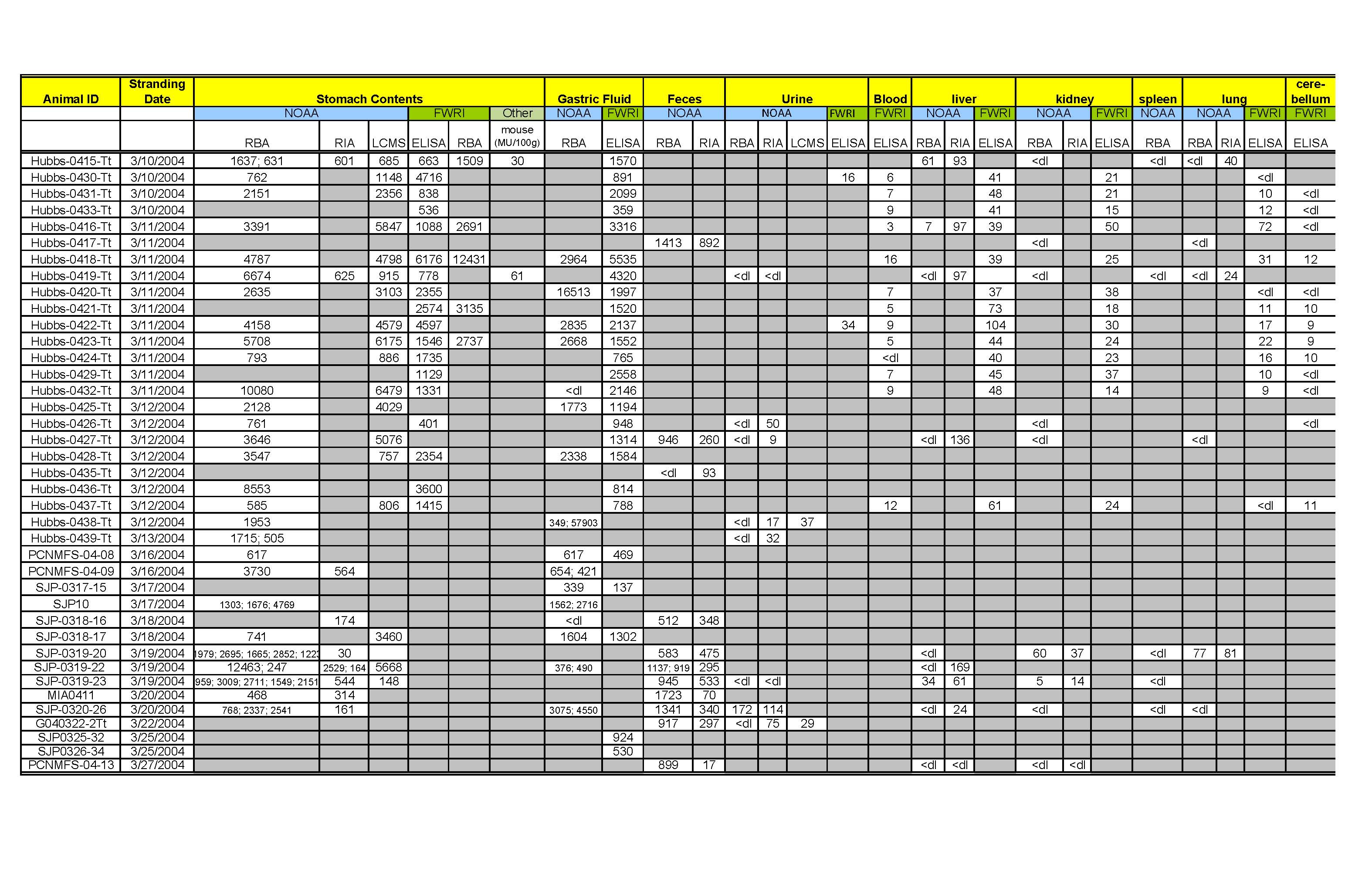
*
